# Persistent pain management in an oncology population through pain neuroscience education, a multimodal program: PaiNEd randomized clinical trial protocol

**DOI:** 10.1371/journal.pone.0290096

**Published:** 2023-08-15

**Authors:** Miguel Ángel Fernández-Gualda, Patrocinio Ariza-Vega, Mario Lozano-Lozano, Irene Cantarero-Villanueva, Lydia Martín-Martín, Eduardo Castro-Martín, Manuel Arroyo-Morales, Isabel Tovar-Martín, Maria Lopez-Garzon, Paula Postigo-Martin, Ángela González-Santos, Francisco Artacho-Cordón, Lucía Ortiz-Comino, Noelia Galiano-Castillo, Carolina Fernández-Lao

**Affiliations:** 1 Health Sciences Faculty, University of Granada, Granada, Spain; 2 Instituto de Investigación Biosanitaria Ibs. GRANADA, Granada, Spain; 3 Department of Physical and Sport Education, PA-HELP "Physical Activity for HEaLth Promotion" Research Group, Faculty of Sports Sciences, University of Granada, Granada, Spain; 4 Sport and Health Research Center (IMUDs), Granada, Spain; 5 Radiation Oncology Department, Virgen de las Nieves University Hospital, Granada, Spain; 6 Department of Radiology and Physical Medicine, University of Granada, Granada, Spain; 7 Health Sciences Faculty (Melilla), University of Granada, Granada, Spain; Maria Sklodowska-Curie National Research Institute of Oncology Krakow, POLAND

## Abstract

**Introduction:**

Pain is one of the most persistent symptoms after cancer treatment. The central nervous system can erroneously stay in its alarm phase, altering the pain experience of patients who have cancer. Pain neuroscience education (PNE) with multimodal approaches may benefit these patients.

**Objective:**

This protocol aims to determine the effectiveness of a PNE tool on pain, physical function and quality of life, as a supplement to a multimodal rehabilitation (MR) program in patients who had breast cancer (BC).

**Methods:**

An 8-week double-blinded randomized controlled trial will be conducted, including 72 participants who had BC and who have persistent pain, randomized into three groups: PNE program + MR program, traditional biomedical information + MR program and control group. The PNE program will include educational content that participants will learn through a mobile app and the MR program will include a concurrent exercise program and manual therapy. The primary outcome will be the perceived pain assessed using the Visual Analogue Scale and secondary outcomes are others related to pain, physical function and quality of life. All outcomes will be evaluated at baseline, at the end of the intervention and 6 months after the end of intervention.

**Discussion:**

The proposed study may help BC patients with persistent pain improve their pain experience, quality of life and provide for more adaptive pain-coping strategies. This protocol could propose an action guide to implement different integral approaches for the treatment of sequelae. This treatment option could be offered to this patient profile and it could be easily implemented in the healthcare systems due to its low costs.

**Trial registration:**

ClinicalTrials.gov, NCT04877860. (February18, 2022).

## Introduction

Despite the increasing incidence [1], more and more patients are surviving cancer due to developments in diagnosis and treatment [2]. However, survivorship does not mean to be illness-free. Persistent/chronic pain is defined as pain that persists for 3 months or longer [2]. Pain is one of the most frequent symptoms after cancer treatment. The burden of persistent pain can be overwhelming in breast cancer (BC) population which is already coping with a cancer diagnosis and treatment-related effects [3]. Moreover, incidence of persistent pain remains high, ranging between 11 to 57% in patients with BC [4].

The main therapeutic agents in cancer are chemotherapy, targeted therapy, immunotherapy, hormonal therapy, surgery, and radiotherapy. Chemotherapy may induce peripheral neuropathy causing neuropathic pain persisting for weeks or years post treatment [5]. Additionally, patients taking aromatase inhibitors to improve their overall survival are at risk of discontinuation during the first 12 months partly due to increased joint pain [6]. Up to 36% of survivors of BC taking aromatase inhibitors can suffer from arthralgia and joint pain [7]. Pain perceived by cancer survivors is often attributed to inflammation resulting from surgical procedures and radiation-induced fibrosis on affected tissues. In terms of shoulder function, a reduced range of motion and loss of strength related to persistent shoulder pain are common adverse effects in patients with BC [8].

Central sensitization (CS) as a new diagnosis label [9], is often accompanied by spreading pain, which extends outside the segmental area of primary nociception [10]. In some cases, after tissues have healed, the central nervous system (CNS) stays erroneously in its alarm phase [9]. This may be altering survivors’ of BC experience of pain, causing an increased responsiveness of nociceptive neurons in the CNS [10]. Apart from hyperalgesia, sensitivity can be altered to environmental stimuli such as weather, light, cold/heat, stress, food [11], and chemical stimuli (pesticides, odors) [12] in this CS context. Both peripheral and CS mechanisms have been identified in patients with BC [13].

Manual therapy may have immediate effect on cancer pain and may improve physical function [14]. Physical exercise enhances side effects derived from cancer treatment, such as fatigue, impaired physical function and pain [15]. Nevertheless, patients with persistent pain have more catastrophic thoughts and fewer adaptive coping strategies so might need a more biopsychosocial explanation of pain [16].

Educational interventions on pain management have mostly centered on traditional biomedical information (TBI), analgesics and explanations of perceived pain were based on tissue issues [17]. The recent paradigm shift in the conception of pain processing has led to pain neuroscience education (PNE) [18]. PNE consists of explaining the neurobiology and neurophysiology of pain and how pain is processed by the nervous system through the influence of stress, emotions, thoughts, sleep quality and various feelings. These biopsychosocial factors become even more important given that cancer patients must deal with a new body image, and possible sexual dysfunction [18]. Introducing PNE is important in cancer patients with persistent pain to decrease patients’ rumination about painful sensations and improve their perceived inability to control pain [19]. PNE in conjunction with other known physiotherapy interventions could ameliorate the prevention and treatment of pain related to medical treatment of cancer.

PNE has previously been used in the physiotherapy management of breast cancer patients with controversial results. Giving PNE instead of biomedical education can be more effective in reducing CS-related symptoms, pain catastrophizing, pain intensity, and pain interference [20]. However, the EduCan trial did not obtain better course of pain-related disability or pain intensity compared with biomedical pain education [21]. In addition, PNE and other physical recovery programs has no side effects [20, 22] and educational contents could be consulted on demand from any smartphone or tablet.

Some previous proposals have monitored and implemented therapeutic recommendations with mobile apps in health problems of high prevalence and high cost to our society, such as hip fractures [23] or the implementation of a recommendation system on energy balance (physical exercise and nutrition) [24] with promising success, both scientifically and socially.

The main aim is to study the short- and long-term effects of a PNE tool as a supplement to a multimodal rehabilitation (MR) program in BC patients with persistent pain upon finishing adjuvant treatment to determine its impact on pain, physical function and quality of life. Our hypothesis is that a PNE tool as a supplement to a MR program based on exercise and manual therapy techniques, will be more effective in improving persistent pain in cancer patients than an MR program combined with TBI. This will be compared to a control group.

## Methods

### Design

**Fig 1:**
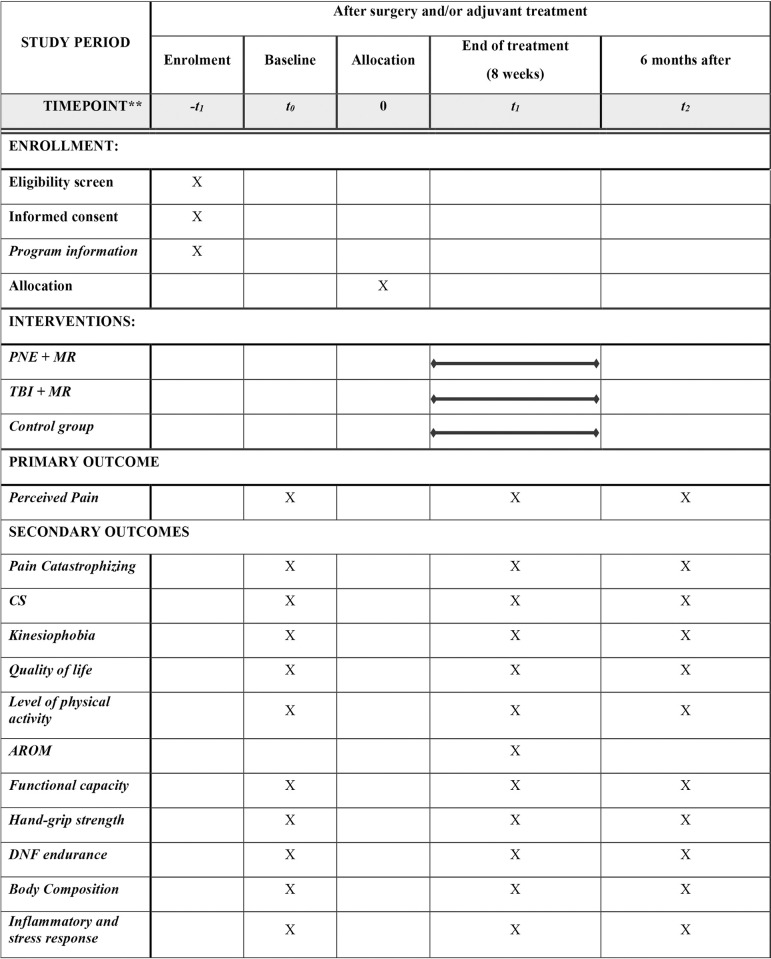
Schedule of enrollment, interventions and assessment following the SPIRIT diagram. AROM: Active range of motion; CS: Central sensitization; DNF: Deep neck flexor; MR: Multimodal rehabilitation; PNE: Pain neuroscience education; TBI: Traditional biomedical information.

The protocol has been developed following the recommendations of the SPIRIT checklist. The study was approved by the Ethics Committee of the Junta de Andalucía (2205-N-20) in concordance with the Declaration of Helsinki. The PaiNEd trial was registered with ClinicalTrials.gov. ID: NCT04877860. A parallel three-arm superiority randomized controlled trial with assessors and data analyzers blinded to the interventions will be performed. Patients will be assessed at three different times, following the recommendations of the SPIRIT checklist and diagram (Fig 1): baseline (t_0_), at the end of the 8-week intervention (t_1_) and 6 months after the end of the intervention (t_2_). Assessments will be performed in 1 session of approximately 2 hours or two different sessions of 1 hour each.

**Fig 2:**
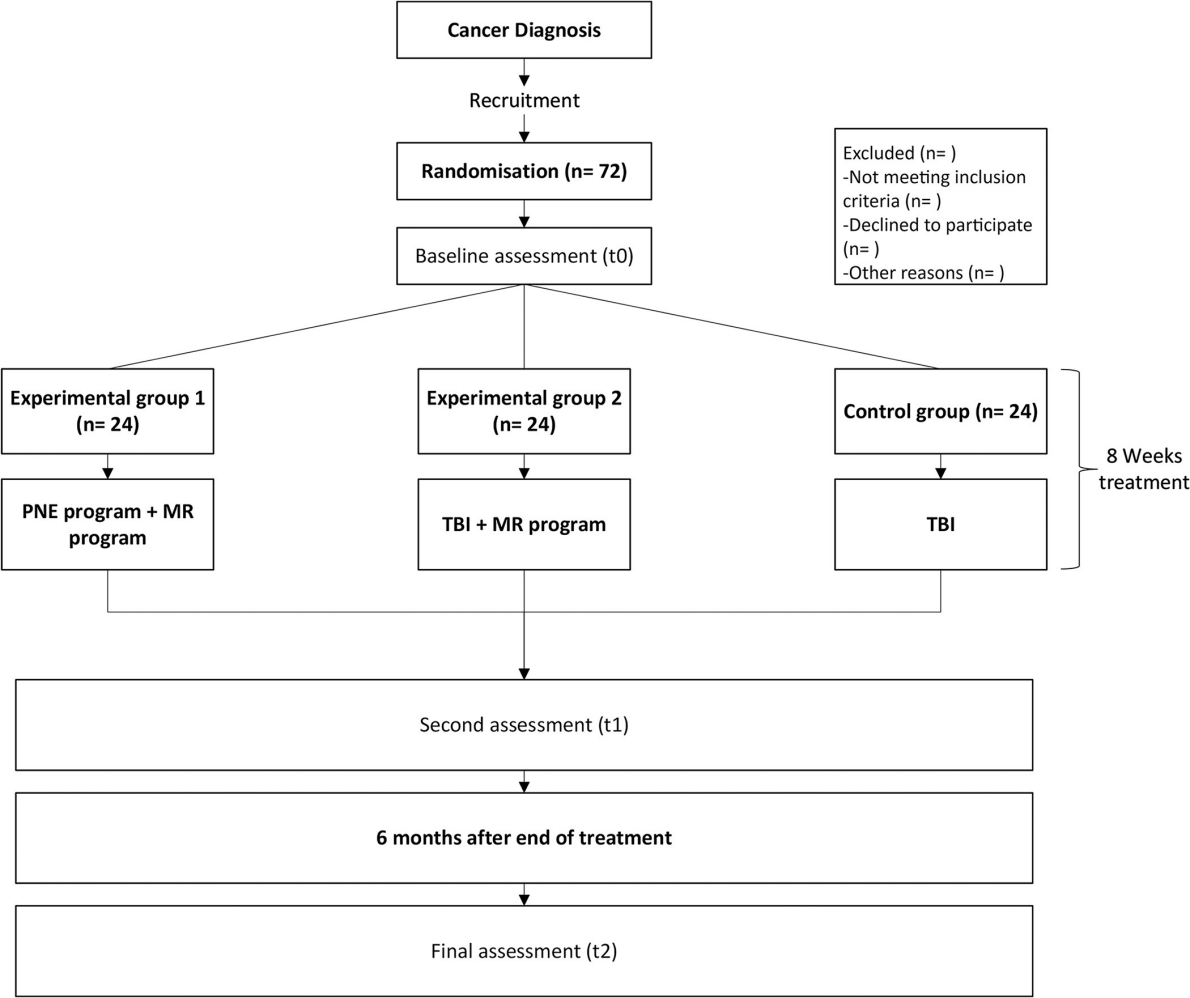
Overview of the study.

Participants will be randomized into three arms (Fig 2). It will take about 2 years to collect and analyze all data. Participants will be recruited from the radiotherapy and oncology services of the "San Cecilio" University Hospital and the "Virgen de las Nieves" University Hospital, Granada, Spain. Interventions will take place at the CUIDATE unit (http://csaludable.ugr.es/pages/dossierultimo/%21), a cancer rehabilitation research unit of the Mixed University Sport and Health Institute, University of Granada.

The inclusion and exclusion criteria for eligible participants of the study are detailed in Table 1.

**Table 1:** Inclusion/Exclusion criteria.

Inclusion criteria	Exclusion criteria
a) Survivors of BC cancer [25]	a) Physical or mental impossibility of carrying out the tests of the study
b) Aged 18 years or older	b) Have suffered chronic pain in the orofacial, cranial, cervical, brachial and shoulder areas or trauma to these regions prior to the cancer diagnosis
c) Have undergone surgery and/or have finished adjuvant treatment (radiotherapy and/or chemotherapy) at least 6 months up to 2 years ago	c)Participating in other physical recovery programs during the interventions
d) Have no active cancer	
d) Have pain ≥ 4 (VAS 0 to 10) in regions related to the tumor area (orofacial, cranial, cervical, brachial and shoulder) for more than 3 months. e) Pain can be compatible with one or more of these conditions: central sensitization, neuropathy, peripheral pain and pain from surgery	

BC = Breast cancer; VAS = Visual analogue scale.

### Randomization and blinding

Upon completion of the recruitment process, participants will be randomized (ratio 1:1) into each of the groups using a random number generation program (EPIDAT 4.2, Xunta de Galicia, Spain). Assessors and data analyzers will be blinded to the randomization of the participants. The blinding of assessors will be guaranteed by the fact that some members of the research group will perform first assessments and others will perform the post-treatment and follow-up assessments.

### Calculation of the sample size

The estimation of the sample size and statistical power for the trial were determined based on the main variable, pain measured through the VAS. Based on a previous study [26] and estimating that patients in the intervention group would have a difference of 15 mm or more in the VAS compared to the control group, differences of at least 5% can be detected with a statistical power of 90% and a significance level alpha α of 0.05. This will require a sample of 24 patients per group, which will result in a total of 72 participants. Therefore, a maximum monitoring loss of 20% will be allowed. To carry out this sample calculation, the G*Power v. 3.1 Software was used.

### Intervention

The PaiNEd study will consist of 24 sessions lasting 8 weeks. The first intervention group will receive the PNE program and MR program. The second group will receive the MR program and TBI, provided in a pamphlet, on the management of pain and disability associated with side effects of cancer treatment. Finally, a control group will receive only the leaflet given to the second group.

#### First arm: PNE program + MR program. (PNE + MR)

For 8 weeks, patients will attend 60 minutes of supervised exercise and one manual therapy session with a duration of 30 minutes every 2 weeks.

#### Second arm: TBI + MR program (TBI+MR)

This intervention group will attend the same multimodal group as the previous one, but without access to the PNE intervention and receiving TBI in a leaflet format.

#### Third arm: Control group

This group will only receive TBI on the management of pain and disability associated with side effects of cancer treatment handed in a leaflet format. Once the measures are registered, they will be invited to participate in the multimodal program for ethical and moral reasons.

#### PNE program

**Fig 3:**
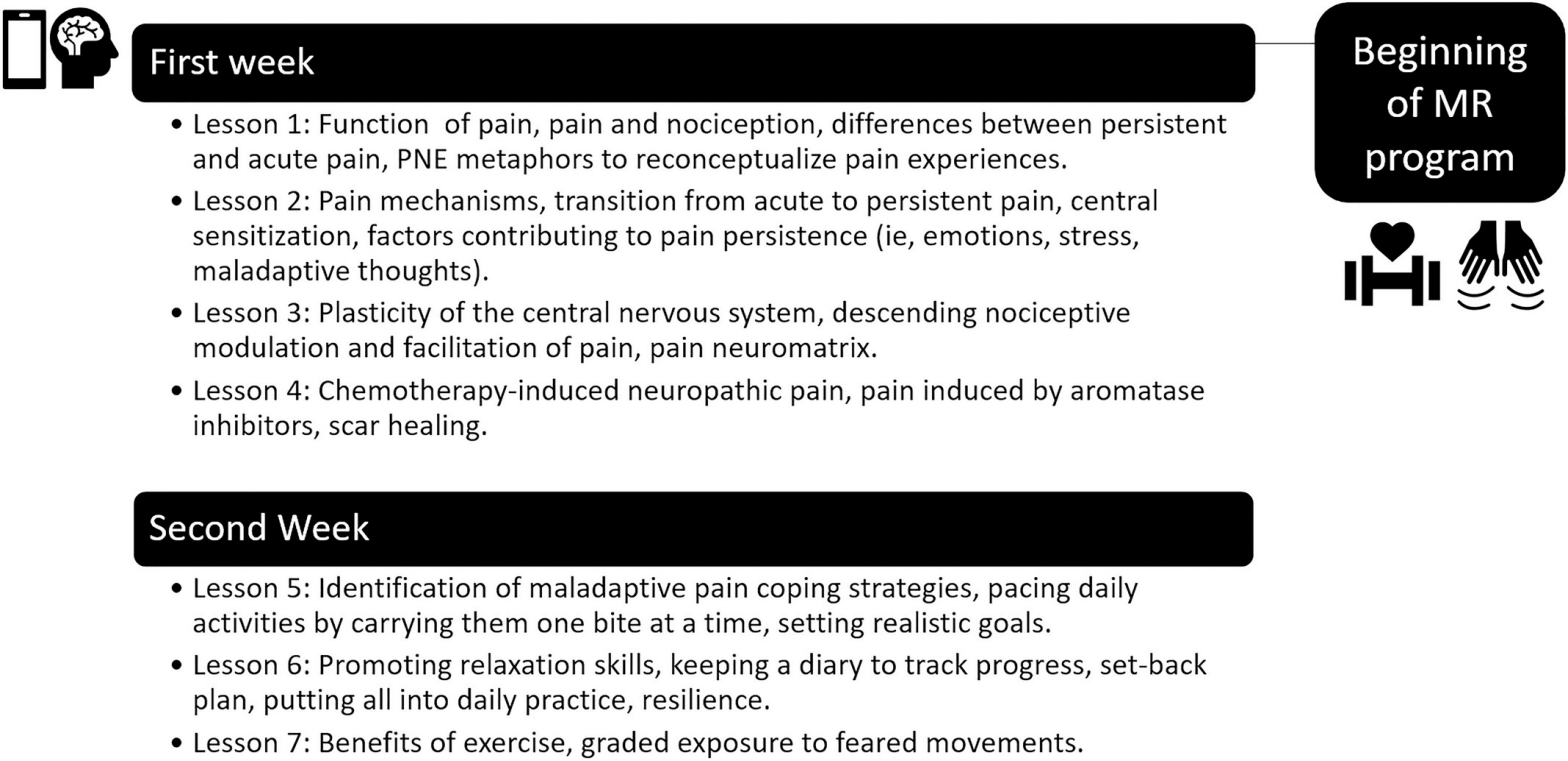
Overview of the PNE program.

This modern PNE program is tailored to the pain needs of survivors of cancer, and the educational content is based on the book “Explain Pain” [18], the Pain In Motion website (http://www.paininmotion.be) and ‘The Pain Toolkit’ [27]. Participants will acquire PNE contents at home during the first two weeks [28] of the MR program. PNE sessions will be delivered to the patients through the PaiNEd app, developed by the BIO277, CUIDATE research group. The app will display lessons following this order (Fig 3), lasting 20 minutes each one. Participants will need a mobile device or tablet with an internet connection and Android or iOS operating systems. They will have access to the app to learn and review the educational contents at home on demand (in video and infographic format).

#### Multimodal rehabilitation program

The MR program features supervised exercise and manual therapy. It will be fully supervised by a physiotherapist who specializes in the implementation of exercise programs and manual therapy in the oncological population and will be individualized to the needs and circumstances of the participants.

*Exercise*. Patients will receive 3 nonconsecutive sessions per week at the CUIDATE unit, and the program will last 8 weeks. An overview of the exercise program is presented in Table 2. The combined resistance and aerobic exercise program will follow the American College of Sports Medicine (ACSM) exercise guidelines [29]. The first and third sessions of the week will consist of 60 minutes of concurrent exercise [30], and the second session will feature 60 minutes of aerobic exercise.

**Table 2:** Overview of the exercise program.

Principles		Concurrent exercise	
	Aerobic exercise		Resistance exercise
Frequency	3 sessions/week		2 sessions/week
Intensity	40–60% HRR		50–70% RM [32]
	Borg CR10 RPE: 3–4 [31]		Borg CR10 RPE: 5–7
Time	Session 1 of the week: 30 minutes		Session 1 of the week: 30 minutes
	Session 2 of the week: 60 minutes		Session 3 of the week: 30 minutes
	Session 3 of the week: 30 minutes		
Type	Elliptical cross-trainer exercise		Squats, stiff-legged deadlifts, shoulder press, unilateral rows, reversed flies and push-ups
			Week 1 to 5: 2x12-20 reps
			Week 6 to 8: 3x10-12 reps
Progression	40–50% HRR; Borg CR10 RPE: 3 (weeks 1 to 4)		When a participant can perform 2 sets, in 2 consecutive sessions with appropriate form, the exercise will be progressed through increasing the resistance by the smallest available increment
	50–60% HRR; Borg CR10 RPE: 4 (weeks 5 to 8) [33]		

HRR: Heart rate reserve; RM: Repetition maximum; RPE: Rating of perceived exertion

*Manual therapy*. Participants will receive 1 manual therapy session every 2 weeks. Each session will last approximately 30 minutes. Treatment sessions will be developed based on previously published protocols that demonstrate effectiveness in treating pain and dysfunction in oncology patients [33, 34]. The current protocol will involve manual and myofascial techniques focusing on the cranio-cervical, thorax and shoulder regions, adapted to the patient´s tissue response [35].

#### Outcomes

All participants will receive written and verbal informed consent and a signed copy of the consent form with the Principal Investigator’s contact details if they have more questions, previous to first assessment. Participants are informed that they can leave the study at any time without penalty or loss of benefits. Eligible participants will not suffer any potential harm in the study. There are no economic incentives for participating in the study. If significant changes regarding objectives, eligibility criteria, outcomes or interventions occur, a formal amendment to the protocol will be needed. Therefore, approval by the Ethics Committee of the Junta de Andalucía and the research team will be required. Clinical data will be taken from the electronic medical records by the participating health personnel. A questionnaire based on sociodemographic issues will be provided at the beginning of the assessment. Questionnaires are collected through the PaiNEd app.

#### Primary outcome (Perceived pain)

The visual analog scale (VAS), offers a subjective pain estimation. The VAS is a 100 mm straight line starting with 0 that represents “no pain” and ends with 100 representing “worst pain imaginable” [36]. The VAS has been used in multiple situations and has proven to be a reliable and appropriate tool to measure pain [36].

#### Secondary outcomes

*Pain catastrophizing*. The Pain Catastrophizing Scale (PCS) is a self-administered scale of 13 items and one of the most used tools to assess catastrophizing in the face of pain. Patients take as a reference their past painful experiences and indicate the level to which they experienced each of the 13 thoughts or feelings in a Likert scale of 5 points that goes from 0 (never) to 4 (always). A total score is obtained from the scale that reflects the level of catastrophizing in the face of the patient’s pain. It contains 3 aspects: a) rumination (constant worry and inability to inhibit thoughts related to pain); b) magnification (the exaggeration of the unpleasantness of situations of pain and expectations of negative consequences), and c) helplessness (the inability to face painful situations). The values of the instrument range between 13 and 62, indicating low scores, low catastrophizing, and high values, high catastrophizing [37]. The PCS has proven to be reliable for daily use in participants reporting chronic pain [37].

*Central sensitization*. The Central Sensitization Inventory (CSI) is composed of two different parts. The first one consists of 25 statements, each answer is scored: 0 Never, 1 Rarely, 2 Sometimes, 3 Often, and 4 Always. So that, at most we will be able to obtain a score of 100 and a minimum of 0. The second part is a survey in which patients explain if they have been diagnosed of specific disorders. Higher CSI scores mean greater symptoms of CS. If patients obtain a score greater than or equal to 40, we will consider that they have sufficient psychometrics to be correlated with CS syndromes [38]. The CSI has shown to be a valid and reliable measure for detecting CS symptoms in survivors of BC [38].

*Kinesiophobia*. Kinesiophobia will be measured by an 11-item version of the Tampa Scale of Kinesiophobia (TSK-11). Each item has 4 response options, “strongly disagree” scores 1 point, and “strongly agree” scores 4 points. The maximum possible score is 44 points and the minimum is 11 points. Greater scores mean strong fear of movement. The Spanish version showed good reliability and validity [39].

*Quality of life*. The European Organization for Research and Treatment of Cancer (EORTC). Created a general quality of life questionnaire known as EORTC–C30 [40]. It is a multidimensional, self-administered questionnaire that consists of 30 questions (Likert type) that is used for all cancer patients (qlq-c30). Global quality of life, functionality (physical, role, emotional, cognitive, and social functioning) and symptomatology (fatigue, nausea and vomiting, pain, dyspnea, insomnia, appetite loss, constipation, diarrhea, and financial difficulties) are evaluated [41]. When analyzing the EORTC questionnaires, scores are obtained in a range from 0 to 100. On the functional items, a higher score means better functioning, and on the symptom items, a higher score turns into a greater presence of the adverse effects. The EORTC QLQ-C30 had an adequate test–retest reliability with an ICC ranging from 0.698 to 0.926 in cancer survivor patients [41].

The QLQ BR-23 module for BC [42], consist of 23 questions covering symptoms and secondary effects related to the different treatment modalities; also includes aspects such as body image, sexuality and future prospects [43]. The BR-23 module has a Cronbach’s α coefficient of 0.873 [44].

*Level of physical activity*. The International Physical Activity Questionnaire (IPAQ) is one of the most used generic physical activity (PA) questionnaires [45]. The short form of IPAQ (IPAQ-SF) consists of 7 questions about the frequency, duration and intensity of PA (vigorous and moderate) performed the last 7 days, as well as walking and sitting time on a working day. Weekly PA is obtained with the calculated MET-minutes within each PA intensity level MET score (Walking  =  3.3 METs, Moderate Physical Activity  =  4.0 METs and Vigorous Physical Activity  =  8.0 METs) [46]. The PA level is classified as ‘high’ (>1500 METs), ‘moderate’ (600–1500 METs), or ‘low’ (<600 METs). The IPAQ-SF has demonstrated acceptable validity in an adult Spanish population [47]. ICC = 0.64; (0.55–0.72) [48].

*Active Range of Motion (AROM)*. A plastic universal goniometer with two adjustable overlapping arms is used to objectively measure the active range of motion of the shoulder joint with the patient placed in the supine position [49]. A cervical range of motion device (Performance Attainment Associates©, Spine Products, Roseville, MN, USA) is used to objectively measure the active range of motion of the cervical joints (sitting position and feet completely in contact with the floor) [50]. AROM measurements are bilaterally performed three times, and the average values are used for the study. Reliability of shoulder AROM was high in survivors of BC, with ICC values ranging from 0.66–0.97. [51]. Cervical AROM is reliable and valid for evaluating the cervical AROM (ICC range: 0.82–0.96) in patients with neck pain [52].

*Functional capacity*. The Six-Minute Walk Test (6MWT) determines the maximum distance (meters) that a person can walk in 6 minutes. The test take place in a 30-metre stretch of unimpeded walkway. Blood pressure, blood oxygen saturation and Borg’s modified Scale (0–10) of RPE are provided at the beginning and the end of the test. Heart rate is measured during the test. Patients are notified when each minute passes and standardized encouragement is given during the test [53]. The test has proven to be reliable as it obtained an ICC of 0.93 in cancer patients [54]. Minimal important difference was established between 22 m (95% CI 18–26) and 32 m (95% CI 20–42) [55].

*Hand-grip strength*. Grip strength is assessed with a manual dynamometer (TKK 5101 Grip-D; Takey, Tokyo, Japan) [56]. The test can be done with the patient in standing position. The elbow is fully extended, the forearm neutral for pronosupination and wrist extension between 0 and 30°. Patients are asked to squeeze the dynamometer as hard as possible. Phrases of encouragement are given to patients in order to achieve their maximum force. The test is repeated three times bilaterally to obtain the mean of each grip. ICC scores range from 0.88 to 0.96 demonstrating high reliability in survivors of HNC [57].

*Deep Neck Flexor (DNF) endurance*. The DNF endurance test measures the endurance of the deep flexor muscles of the neck [58]. It consists of placing the patient in supine position with the spine fully supported on the stretcher and asking him to first, to do a flexion of the upper cervical spine and to simultaneously flex the lower cervical spine. The time that the patient is able to maintain the position without losing the initial distance that separated him from the stretcher is counted. The test will stop if the participant reports pain or if he can no longer maintain the position. Reliability of DNF endurance test was moderate as ICC = 0.66 [59].

*Body composition*. Direct Segmental Multi-frequency Bioelectrical Impedance Analysis (DSM-BIA) is used for assessing skeletal muscle mass (Kg), fat mass (%) and body weight (kg). DSM-BIA is evaluated using the In-Body 720 body composition analyzer (Biospace, Seoul, Korea), following the instructions of the user’s manual [60]. Patients will be told not to eat or drink one hour before the test. BIA appeared to have very high reliability at estimating body fat percentage in survivors of BC as ICC = 1 [61] and 0.95 at estimating muscle mass in women [62].

### Inflammatory and stress response

Saliva will be collected in the morning period (09:00 to 11:30 a.m.) [63]. The salivary biomarkers Cortisol and Interleukin 6 (IL6) will be measured with a commercial luminescence immune assay (Salimetrics, State College, PA) and the Salimetrics IL-6 ELISA Kit respectively. Cortisol is modulated by chronic stress generated by the constant pain endured by the patients [64]. IL6 is the cytokine which regulates inflammatory response to physical or psychological (affective) alteration of the organism. Some precautions must be taken for its collection, as participants will be instructed to not brush their teeth for at least 30 minutes before and to avoid eating, drinking and performing PA at least 3 hours before.

### Data collection and security

To enhance the retention of treatment groups, the app will show users when they come to the cancer rehabilitation research unit for assessments or multimodal treatment sessions, as well as how many weeks and sessions remain. Participants will also be notified of their assessment and treatment appointments by phone or e-mail according to their preferences.

The mobile app will be installed in each patient’s mobile and instructions about how properly use it will be given. All data will be stored in accordance with the European General Data Protection Regulation.

Only the researchers involved in the PaiNEd protocol will have physical access to all pseudoanonymized and encrypted information, with the server located at the University of Granada (Granada, Spain). This will ensure the safety of data management. Personal data will be handled in a way that they can no longer be attributed to study participants, following the principles of anonymity and confidentiality throughout the study.

### Data analysis

To analyze the results, the statistical program IBM SPSS 26 (IBM Corp., Armonk, NY, USA.) will be used. The descriptive statistics for this study (95% confidence intervals) will use mean and standard deviation (continuous data) or frequencies and percentages (categorical data). The Kolmogorov-Smirnov test will be applied to verify the normal distribution of the variables (p> 0.05). Receiver operating characteristic (ROC) curves will be included to determine the accuracy of the tests. A t test will be used to analyze parametric variables, and a Mann–Whitney U test will be used for nonparametric variables. From the point of view of inferential statistics and to respond to the main objective of this study, an intention-to-treat analysis will be carried out. A 3x3 repeated-measures ANOVA model will be performed using time (baseline, postintervention, follow-up) as a within-subject factor and intervention (first arm, second arm, control) as a between-subjects factor. The Bonferroni test will be used for post hoc analysis. For the study on subgroups with possible differential results, cancer location, type of treatment, elevated tumor stages, age, sex will be used as possible confounding variables in a subsequent multiple regression analysis. A significance level of p value less than 0.05 (P < .05) will be considered. The intergroup effect sizes will be calculated to provide magnitude changes; the effect size will be estimated using Cohen’s d (0–0.19, negligible; 0.20–0.49, small; 0.50–0.79, moderate; ≥0.8, large).

## Discussion

The current trial will determine if the application of combined PNE with an MR program presents higher efficacy in reducing pain than the application of this MR program without PNE. Previous studies have demonstrated that the combination of PNE with other therapies such as exercise or dry needling, obtains better results in kinesiophobia and pain than the sum of its parts in chronic neck pain [65, 66]. These findings in PNE are consistent with the results obtained in chronic low back pain and other chronic musculoskeletal pain conditions, in conjunction with usual physiotherapy interventions and exercise, respectively [67, 68]. However, despite these benefits, to our knowledge no intervention has tested the effects of a PNE app within a physical therapy program in a breast cancer population suffering from pain and dysfunction. Furthermore, earlier evidence shows that more research is needed to observe the effects of pain management and healthy lifestyle interventions in cancer survivors [69].

Different studies have shown the efficacy of MR programs (exercise, kinesitherapy and/or manual therapy) in health issues related to pain and dysfunction associated with medical treatment of BC [70–72] or colon cancer [73]. We therefore consider it possible that similar results could be achieved with MR programs in patients who had breast cancer and present with persistent pain. A recent study has piloted the effectiveness of a PNE session in cancer survivors, and they present preliminary but promising results [19]. It seems necessary to design and implement appropriate treatment programs for this specific population. Currently, the cancer population still faces difficulties in receiving optimal treatment programs for their illness or survivorship heaviness and support to maintain a healthy lifestyle [69].

Although participants can also have different levels of PA outside the program. It will be registered with the IPAQ-SF questionnaire and considered for analyses. Even though low adherence levels might be expected from the exercise program, supervision by the physical therapist and social interaction between participants will reinforce adherence and physical function. This could open up the possibility to participants to share their pain coping strategies and all achievements made. In addition, despite there will be participants who will not know how to use the app, they will be taught how to properly use it to benefit the most from PNE.

The proposed study may help BC patients with persistent pain improve their pain experience, quality of life and provide for more adaptive pain-coping strategies. This protocol could propose an action guide to implement different integral approaches for the treatment of sequelae. If the group that includes PNE sessions proves to be more effective in reducing pain and improving physical function and quality of life than the groups without PNE, this treatment option could be offered to this patient profile and it could be easily implemented in the healthcare systems due to its low costs. Our results will be disseminated through social media, professional conferences and international refereed journals.

We expect moderate effectiveness on pain, physical function and quality of life due to the multimodal approach and the fact that we give the PNE contents 6 months after having undergone surgery and/or have finished adjuvant treatment (radiotherapy and/or chemotherapy). Providing PNE in the early postoperative stage [21], could be a barrier in the process of reconceptualizing pain as having psychological distress and poor cognitive functions (attention and memory) are common in that period [74–76].

Results from the PaiNEd trial will be disseminated in peer-reviewed journals, national and international conferences focused on pain, oncology and rehabilitation. Furthermore, findings will also be available through social media of the research team. Finally, participants and other interested cancer patients will know the final results of the study.

## Supporting information

S1 Checklist SPIRIT 2013 checklist: Recommended items to address in a clinical trial protocol and related documents*. (PDF)

S2 Checklist SPIRIT 2013 checklist: Recommended items to address in a clinical trial protocol and related documents*. (PDF)

S1 File (PDF)

S2 File (PDF)
