# Supplementary material for: Persistent pain management in an oncology population through pain neuroscience education, a multimodal program: PaiNEd randomized clinical trial protocol
Source: PLoS One. 2023 Aug 15;18(8):e0290096. doi: 10.1371/journal.pone.0290096 (PMC10426993; doi:10.1371/journal.pone.0290096)
Supplement: S1 Checklist — (PDF) [file pone.0290096.s001.pdf]

SPIRIT 2013 Checklist: Recommended items to address in a clinical trial protocol and related documents*

Section/item	Item No	Description
**Administrative information**		
Title	1	Descriptive title identifying the study design, population, interventions, and, if applicable, trial acronym **(Page number 1, page number 8)**
Trial registration	2a	Trial identifier and registry name. If not yet registered, name of intended registry **(Page number 9, page number 12)**
	2b	All items from the World Health Organization Trial Registration Data Set **(Page number 9, page number 12)**
Protocol version	3	Date and version identifier **(Page number 9)**
Funding	4	Sources and types of financial, material, and other support **(Submission system)**
Roles and responsibilities	5a	Names, affiliations, and roles of protocol contributors **(Page number 2 to 7)**
	5b	Name and contact information for the trial sponsor **(Submission system)**
	5c	Role of study sponsor and funders, if any, in study design; collection, management, analysis, and interpretation of data; writing of the report; and the decision to submit the report for publication, including whether they will have ultimate authority over any of these activities **(Submission system)**
	5d	Composition, roles, and responsibilities of the coordinating centre, steering committee, endpoint adjudication committee, data management team, and other individuals or groups overseeing the trial, if applicable (see Item 21a for data monitoring committee) **(Submission system)**
**Introduction**		
Background and rationale	6a	Description of research question and justification for undertaking the trial, including summary of relevant studies (published and unpublished) examining benefits and harms for each intervention **(Page number 10 to 12)**
	6b	Explanation for choice of comparators **(Page number 10 to 12)**

Objectives	7	Specific objectives or hypotheses **(Page number 12)**
Trial design	8	Description of trial design including type of trial (eg, parallel group, crossover, factorial, single group), allocation ratio, and framework (eg, superiority, equivalence, noninferiority, exploratory) **(Page number 12)**

Methods: Participants, interventions, and outcomes

Study setting	9	Description of study settings (eg, community clinic, academic hospital) and list of countries where data will be collected. Reference to where list of study sites can be obtained **(Page number 13)**
Eligibility criteria	10	Inclusion and exclusion criteria for participants. If applicable, eligibility criteria for study centres and individuals who will perform the interventions (eg, surgeons, psychotherapists) **(Page number 13)**
Interventions	11a	Interventions for each group with sufficient detail to allow replication, including how and when they will be administered **(Page number 14 to 17)**
	11b	Criteria for discontinuing or modifying allocated interventions for a given trial participant (eg, drug dose change in response to harms, participant request, or improving/worsening disease) **(Page number 13)**
	11c	Strategies to improve adherence to intervention protocols, and any procedures for monitoring adherence (eg, drug tablet return, laboratory tests) **(Page number 24)**
	11d	Relevant concomitant care and interventions that are permitted or prohibited during the trial **(Page number 24)**
Outcomes	12	Primary, secondary, and other outcomes, including the specific measurement variable (eg, systolic blood pressure), analysis metric (eg, change from baseline, final value, time to event), method of aggregation (eg, median, proportion), and time point for each outcome. Explanation of the clinical relevance of chosen efficacy and harm outcomes is strongly recommended **(Page number 17 to 21)**
Participant timeline	13	Time schedule of enrolment, interventions (including any run-ins and washouts), assessments, and visits for participants. A schematic diagram is highly recommended (see Figure) **(Page number 12)**
Sample size	14	Estimated number of participants needed to achieve study objectives and how it was determined, including clinical and statistical assumptions supporting any sample size calculations **(Page number 14)**
Recruitment	15	Strategies for achieving adequate participant enrolment to reach target sample size **(Page number 13, page number 14)**

Methods: Assignment of interventions (for controlled trials)

Allocation:

Sequence generation	16a	Method of generating the allocation sequence (eg, computer-generated random numbers), and list of any factors for stratification. To reduce predictability of a random sequence, details of any planned restriction (eg, blocking) should be provided in a separate document that is unavailable to those who enrol participants or assign interventions **(Page number 14)**
Allocation concealment mechanism	16b	Mechanism of implementing the allocation sequence (eg, central telephone; sequentially numbered, opaque, sealed envelopes), describing any steps to conceal the sequence until interventions are assigned **(Page number 14)**
Implementation	16c	Who will generate the allocation sequence, who will enrol participants, and who will assign participants to interventions **(Page number 14)**
Blinding (masking)	17a	Who will be blinded after assignment to interventions (eg, trial participants, care providers, outcome assessors, data analysts), and how **(Page number 14)**
	17b	If blinded, circumstances under which unblinding is permissible, and procedure for revealing a participant's allocated intervention during the trial **(Page number 14)**

Methods: Data collection, management, and analysis

Data collection methods	18a	Plans for assessment and collection of outcome, baseline, and other trial data, including any related processes to promote data quality (eg, duplicate measurements, training of assessors) and a description of study instruments (eg, questionnaires, laboratory tests) along with their reliability and validity, if known. Reference to where data collection forms can be found, if not in the protocol **(Page number 21 to 22)**
	18b	Plans to promote participant retention and complete follow-up, including list of any outcome data to be collected for participants who discontinue or deviate from intervention protocols **(Page number 21 to 22)**
Data management	19	Plans for data entry, coding, security, and storage, including any related processes to promote data quality (eg, double data entry; range checks for data values). Reference to where details of data management procedures can be found, if not in the protocol **(Page number 22)**
Statistical methods	20a	Statistical methods for analysing primary and secondary outcomes. Reference to where other details of the statistical analysis plan can be found, if not in the protocol **(Page number 22 to 23)**

	20b	Methods for any additional analyses (eg, subgroup and adjusted analyses) **(Page number 22 to 23)**
	20c	Definition of analysis population relating to protocol non-adherence (eg, as randomised analysis), and any statistical methods to handle missing data (eg, multiple imputation) **(Page number 22 to 23)**
**Methods: Monitoring**		
Data monitoring	21a	Composition of data monitoring committee (DMC); summary of its role and reporting structure; statement of whether it is independent from the sponsor and competing interests; and reference to where further details about its charter can be found, if not in the protocol. Alternatively, an explanation of why a DMC is not needed **(Page number 21 to 22)**
	21b	Description of any interim analyses and stopping guidelines, including who will have access to these interim results and make the final decision to terminate the trial **(Page number 22)**
Harms	22	Plans for collecting, assessing, reporting, and managing solicited and spontaneously reported adverse events and other unintended effects of trial interventions or trial conduct **(Page number 22)**
Auditing	23	Frequency and procedures for auditing trial conduct, if any, and whether the process will be independent from investigators and the sponsor **(Page number 22)**
**Ethics and dissemination**		
Research ethics approval	24	Plans for seeking research ethics committee/institutional review board (REC/IRB) approval **(Page number 7, page number 12)**
Protocol amendments	25	Plans for communicating important protocol modifications (eg, changes to eligibility criteria, outcomes, analyses) to relevant parties (eg, investigators, REC/IRBs, trial participants, trial registries, journals, regulators) **(Page number 7, page number 17)**
Consent or assent	26a	Who will obtain informed consent or assent from potential trial participants or authorised surrogates, and how (see Item 32) **(Page number 17)**
	26b	Additional consent provisions for collection and use of participant data and biological specimens in ancillary studies, if applicable **(Page number 17)**
Confidentiality	27	How personal information about potential and enrolled participants will be collected, shared, and maintained in order to protect confidentiality before, during, and after the trial **(Page number 22)**
Declaration of interests	28	Financial and other competing interests for principal investigators for the overall trial and each study site **(Submission system)**

Access to data	29	Statement of who will have access to the final trial dataset, and disclosure of contractual agreements that limit such access for investigators **(Page number 22)**
Ancillary and post-trial care	30	Provisions, if any, for ancillary and post-trial care, and for compensation to those who suffer harm from trial participation **(Page number 17)**
Dissemination policy	31a	Plans for investigators and sponsor to communicate trial results to participants, healthcare professionals, the public, and other relevant groups (eg, via publication, reporting in results databases, or other data sharing arrangements), including any publication restrictions **(Page number 24)**
	31b	Authorship eligibility guidelines and any intended use of professional writers **(Page number 24)**
	31c	Plans, if any, for granting public access to the full protocol, participant-level dataset, and statistical code **(Page number 24)**
**Appendices**		
Informed consent materials	32	Model consent form and other related documentation given to participants and authorised surrogates **Informed consent is provided at the end of this document**
Biological specimens	33	Plans for collection, laboratory evaluation, and storage of biological specimens for genetic or molecular analysis in the current trial and for future use in ancillary studies, if applicable **Not applicable**

*It is strongly recommended that this checklist be read in conjunction with the SPIRIT 2013 Explanation & Elaboration for important clarification on the items. Amendments to the protocol should be tracked and dated. The SPIRIT checklist is copyrighted by the SPIRIT Group under the Creative Commons "Attribution-NonCommercial-NoDerivs 3.0 Unported" license.

HOJA DE INFORMACIÓN AL PARTICIPANTE

Título del estudio: PaiNEd Study: EFECTIVIDAD DE UN SISTEMA DE E-HEALTH INTEGRADO EN UN PROGRAMA DE RECUPERACIÓN FÍSICA PARA EL TRATAMIENTO DEL DOLOR EN POBLACIÓN ONCOLÓGICA.

.

INVESTIGADOR PRINCIPAL/DEPARTAMENTO/EMAIL:

CAROLINA FERNÁNDEZ LAO/FISIOTERAPIA/carolinafl@ugr.es

CENTRO:

UNIDAD DE APOYO AL PACIENTE ONCOLOGICO (GRANADA)

1. INTRODUCCIÓN

Le informaremos acerca de un estudio de investigación, aprobado por el Comité de Ética, en el que está invitado a participar. Nuestra única intención es que reciba la información correcta y suficiente para que pueda evaluar y juzgar si desea o no participar en este estudio. Para hacer esto, lea esta Hoja de Información cuidadosamente. Puede consultar con las personas que considere apropiadas y aclararemos cualquier duda que pueda surgir.

El investigador y las entidades colaboradoras se comprometen a cumplir estrictamente la legislación nacional, regional y de la UE que cubre el uso de datos humanos con fines científicos.

Este proyecto está en concordancia con los Principios éticos fundamentales, incluidos los reflejados en la Carta de los Derechos Fundamentales de la Unión Europea[1] y las reglas éticas relevantes de H2020. Además, se aplican las siguientes normas internacionales:

Declaración de WMA de Helsinki[2]

Convención de Bioética de Oviedo[3]

---

[1] Charter of Fundamental Rights of the European Union, 2000/C 364/01, http://www.europarl.europa.eu/charter/pdf/text_en.pdf

[2] Declaration of Helsinki, http://www.who.int/bulletin/archives/79(4)373.pdf

[3] Convention for the Protection of Human Rights and Dignity of the Human Being with regard to the Application of Biology and Medicine: Convention on Human Rights and Biomedicine, https://rm.coe.int/CoERMPublicCommonSearchServices/DisplayDCTMContent?documentId=090000168007cf98

2.PARTICIPACIÓN VOLUNTARIA

Debe saber que su participación en este estudio es voluntaria, y puede decidir no participar o cambiar su decisión y retirar su consentimiento en cualquier momento. Su participación en el estudio en ningún caso representará una carga financiera adicional para usted.

3.DESCRIPCIÓN GENERAL DEL ESTUDIO

Este proyecto lo llevará a cabo el personal del grupo de investigación llamado CUIDATE y se desarrollará en la Unidad de Apoyo al Paciente Oncológico que surge del convenio establecido entre la Universidad de Granada (Instituto de Investigación Deporte y Salud) y el Servicio Andaluz de Salud. Dicho centro cuenta con los espacios y equipamiento necesarios para completar el programa de intervención y las pertinentes evaluaciones basal, post-tratamiento y tras el seguimiento. Somos un grupo de fisioterapeutas, terapeutas ocupacionales, médicos oncólogos, especialistas en actividad física y especialistas en medicina del deporte que tienen como objetivo mejorar la calidad de vida de los pacientes oncológicos. El grupo pretende evaluar los problemas que le preocupan y darle respuestas de forma individualizada.

Le estamos pidiendo que participe en este estudio porque podrá mejorar su calidad de vida tras haber sufrido cáncer. La quimioterapia forma parte, en la mayoría de los casos, del arsenal terapéutico que reciben los pacientes oncológicos junto con la radioterapia y la cirugía. Entre los efectos secundarios asociados el dolor y la disfunción pueden ser invalidantes para algunas personas y motivo de abono del tratamiento médico. Por ello, queremos ofrecerte una intervención basada en un programa de rehabilitación multimodal (acompañado de un programa de educación), sólo solo rehabilitación multimodal (más una serie de recomendaciones), o una serie de recomendaciones basadas en ejercicios para poder dar respuesta a las necesidades que le surgen después del proceso oncológico y para ayudarle en el camino de su recuperación. El propósito de este estudio es evaluar la efectividad de los tratamientos sobre la calidad de vida. Se está llevando a cabo esta investigación por dos razones fundamentales: son técnicas seguras, específicas para el dolor y la disfunción y coste-efectivas, y además porque no existen estudios que usen esta combinación de tratamientos, aunque de manera aislada ya han mostrado efectividad en otros síndromes de dolor crónico.

Aproximadamente 80 personas participarán en el estudio. Al tratarse de un estudio experimental, se realizarán dos grupos de intervención y un grupo control y ustedes podrán ser asignados de forma aleatoria a uno de los grupos.

Si usted decide participar en este estudio, tendrá que dar respuestas a una serie de cuestionarios sobre su salud y realizar test físicos para analizar la respuesta de su cuerpo al recibir los tratamientos descritos anteriormente.

Permanecerá en el estudio 32 semanas (8 meses) debido al seguimiento que se realizará durante este tiempo. Le realizaremos una valoración basal, al inicio del estudio, y dos valoraciones de seguimiento: una al finalizar la intervención y otra a los 6 meses.

4.BENEFICIOS Y RIESGOS DERIVADOS DE SU PARTICIPACIÓN EN EL ESTUDIO.

Los posibles beneficios de participar en el estudio son al menos los mismos que se obtienen al recibir intervención convencional sin participar en el estudio.

Le notificaremos sobre cualquiera nueva información que pueda afectar a su salud, bienestar o interés por continuar en el estudio.

Con su participación puede ayudarnos a mejorar la información existente, lo que nos permite mejorar la asistencia a las personas que pasan por un proceso similar al suyo. Obtendrá un informe detallado de todas las evaluaciones llevadas a cabo una vez finalizado el proyecto, que verá la evolución de su salud a través de su tratamiento. Por otro

lado, también recibirá pautas sobre manejo del dolor y bienestar psicológico que puede seguir una vez que el proyecto finalice, independientemente del grupo de estudio al que pertenezca.

5. PROTECCIÓN DE DATOS

El procesamiento, la comunicación y la transferencia de los datos personales de todos los participantes se realizarán de conformidad con las reglamentaciones locales, nacionales e internacionales. De acuerdo con la legislación antes mencionada, puede ejercer sus derechos de acceso, modificación, oposición y cancelación de datos, para lo cual debe contactar a su persona de estudio a cargo.

A partir del 25 de mayo de 2018 es de plena aplicación la nueva legislación en la UE sobre datos personales, en concreto el Reglamento (UE) 2016/679 del Parlamento europeo y del Consejo de 27 de abril de 2016 de Protección de Datos (RGPD). Por ello, es importante que conozca la siguiente información.

- Además de los derechos que ya conoce (acceso, modificación, oposición y cancelación de datos) ahora también puede limitar el tratamiento de datos que sean incorrectos, solicitar una copia o que se trasladen a un tercero (portabilidad) los datos que usted. ha facilitado para el estudio. Para ejercitar sus derechos, diríjase al investigador principal del estudio. Le recordamos que los datos no se pueden eliminar aunque deje de participar en el ensayo para garantizar la validez de la investigación. Así mismo tiene derecho a dirigirse a la Agencia de Protección de Datos si no quedara satisfecho/a.
- Tanto el Centro como el Promotor son responsables respectivamente del tratamiento de sus datos y se comprometen a cumplir con la normativa de protección de datos en vigor. Los datos recogidos para el estudio estarán identificados mediante un código, de manera que no se incluya información que pueda identificarle, y sólo su médico del estudio/colaboradores podrá relacionar dichos datos con usted y con su historia clínica. Por lo tanto, su identidad no será revelada a ninguna otra persona salvo a las autoridades sanitarias, cuando así lo requieran o en casos de urgencia médica. Los Comités de Ética de la Investigación, los representantes de la Autoridad Sanitaria en materia de inspección y el personal autorizado por el Promotor, únicamente podrán acceder para comprobar los datos personales, los procedimientos del estudio clínico y el cumplimiento de las normas de buena práctica clínica (siempre manteniendo la confidencialidad de la información).
- El Investigador y el Promotor están obligados a conservar los datos recogidos para el estudio al menos hasta 25 años tras su finalización. Posteriormente, su información personal solo se conservará por el centro para el cuidado de su salud y por el promotor para otros fines de investigación científica si usted hubiera otorgado su consentimiento para ello, y si así lo permite la ley y requisitos éticos aplicables.
- Si realizáramos transferencia de sus datos codificados fuera de la UE a las entidades de nuestro grupo, a prestadores de servicios o a investigadores científicos que colaboren con nosotros, los datos del participante quedarán protegidos con salvaguardas tales como contratos u otros mecanismos por las autoridades de protección de datos.

6. COMPENSACION ECONÓMICA

El investigador del estudio es responsable de gestionar el financiamiento de la misma. El sujeto no recibirá ninguna remuneración. La participación en el estudio no implicará un costo adicional.

7. OTRA INFORMACIÓN RELEVANTE

El grupo de investigación le comunicará lo antes posible cualquier información nueva relevante para el estudio que pueda afectar su disposición a participar en el estudio, que se descubra durante su participación.

Si decide retirar su consentimiento para participar en este estudio, no se agregarán nuevos datos a la base de datos y puede requerir la destrucción de datos anteriores.

También debe saber que puede ser excluido del estudio si los investigadores del estudio lo consideran apropiado, ya sea por razones de seguridad, por cualquier ocurrencia que ocurra o porque consideren que no está cumpliendo con los procedimientos establecidos. En cualquier caso, recibirá una explicación adecuada del motivo de su retirada del

estudio. Al firmar el formulario de consentimiento informado, usted acepta cumplir con los procedimientos de estudio que se han establecido.

CONSENTIMIENTO INFORMADO – CONSENTIMIENTO POR ESCRITO DEL PACIENTE

PaiNEd Study: EFECTIVIDAD DE UN SISTEMA DE E-HEALTH INTEGRADO EN UN PROGRAMA DE RECUPERACIÓN FÍSICA PARA EL TRATAMIENTO DEL DOLOR EN POBLACIÓN ONCOLÓGICA.

Yo (Nombre y Apellidos):..

- He leído el documento informativo que acompaña a este consentimiento (Información al Paciente)
- He podido hacer preguntas sobre el estudio "**PaiNEd Study: EFECTIVIDAD DE UN SISTEMA DE E-HEALTH INTEGRADO EN UN PROGRAMA DE RECUPERACIÓN FÍSICA PARA EL TRATAMIENTO DEL DOLOR EN LA POBLACIÓN ONCOLÓGICA**".
- He recibido suficiente información sobre el estudio ""**PaiNEd Study: EFECTIVIDAD DE UN SISTEMA DE E-HEALTH INTEGRADO EN UN PROGRAMA DE RECUPERACIÓN FÍSICA PARA EL TRATAMIENTO DEL DOLOR EN LA POBLACIÓN ONCOLÓGICA**". He hablado con el profesional sanitario informador: ………………………………………………………………
- Comprendo que mi participación es voluntaria y soy libre de participar o no en el estudio.
- Se me ha informado que todos los datos obtenidos en este estudio serán confidenciales y se tratarán conforme establece la Ley Orgánica de Protección de Datos de Carácter Personal 15/99.
- Se me ha informado de que la donación/información obtenida sólo se utilizará para los fines específicos del estudio.
- **Deseo** ser informado/a de mis datos genéticos y otros de carácter personal que se obtengan en el curso de la investigación, incluidos los descubrimientos inesperados que se puedan producir, siempre que esta información sea necesaria para evitar un grave perjuicio para mi salud o la de mis familiares biológicos.
  Si No

Comprendo que puedo retirarme del estudio:

- Cuando quiera
- Sin tener que dar explicaciones
- Sin que esto repercuta en mis cuidados médicos

Presto libremente mi conformidad para participar en el *proyecto titulado* "**PaiNEd Study: EFECTIVIDAD DE UN SISTEMA DE E-HEALTH INTEGRADO EN UN PROGRAMA DE RECUPERACIÓN FÍSICA PARA EL TRATAMIENTO DEL DOLOR EN LA POBLACIÓN ONCOLÓGICA**".

Firma del paciente	Firma del profesional
(o representante legal en su caso)	sanitario informador
Nombre y apellidos:……………….	Nombre y apellidos: ………..
Fecha: ………………………………	Fecha: ……………………….

REVOCACIÓN DEL CONSENTIMIENTO INFORMADO PARA LA PARTICIPACIÓNEN

EL PROYECTO DE INVESTIGACIÓN

Yo, D./Dª ……………………………………………………, con DNI/NIE……………………………………., como (marcar lo que proceda): SUJETO/ REPRESENTANTE LEGAL, revoco libremente el consentimiento informado para la participación en el proyecto de investigación firmado en el presente documento.

* En este caso, al tener el sujeto participante un impedimento para escribir, la revocación del consentimiento se realiza de forma oral en presencia del testigo D./Dª ………………………………….., con DNI/NIE…………………..., que firma a continuación

**Firma del sujeto/Representante legal/Testigo**	**Firma del investigador y Nº de colegiado**
Fecha	Fecha

PLAN DE CONTINGENCIA

***Estudio* PaiNEd Study: EFECTIVIDAD DE UN SISTEMA DE E-HEALTH INTEGRADO EN UN PROGRAMA DE RECUPERACIÓN FÍSICA PARA EL TRATAMIENTO DEL DOLOR EN POBLACIÓN ONCOLÓGICA.**

La Dra. Carolina Fernández Lao, con D.N.I. 75127340 e I.P. del estudio "PaiNEd Study: EFECTIVIDAD DE UN SISTEMA DE E-HEALTH INTEGRADO EN UN PROGRAMA DE RECUPERACIÓN FÍSICA PARA EL TRATAMIENTO DEL DOLOR EN POBLACIÓN ONCOLÓGICA", se comprometea a la destrucción, si el paciente así lo requiere del excedente de muestras o cesión al Biobanco del SSPA tras la finalización del estudio PaiNEd Study: EFECTIVIDAD DE UN SISTEMA DE E-HEALTH INTEGRADO EN UN PROGRAMA DE RECUPERACIÓN FÍSICA MULTIMODAL PARA EL TRATAMIENTO DEL DOLOR EN POBLACIÓN ONCOLÓGICA, para así estar disponibles para cualquier otro estudio de investigación que las requiera.

En Granada, a 27 de Julio de 2010

Fdo. Carolina Fernández Lao
