# Supplementary material for: Persistent pain management in an oncology population through pain neuroscience education, a multimodal program: PaiNEd randomized clinical trial protocol
Source: PLoS One. 2023 Aug 15;18(8):e0290096. doi: 10.1371/journal.pone.0290096 (PMC10426993; doi:10.1371/journal.pone.0290096)
Supplement: S1 File — (PDF) [file pone.0290096.s003.pdf]

TÍTULO DEL PROYECTO

EFECTIVIDAD DE UN SISTEMA DE E-HEALTH INTEGRADO EN UN PROGRAMA DE RECUPERACIÓN FÍSICA PARA EL TRATAMIENTO DEL DOLOR EN SUPERVIVIENTES DE CÁNCER. PaiNEd STUDY

PALABRAS CLAVE

Cáncer, dolor, secuelas, educación, tratamiento multimodal

1. RESUMEN

Las personas que han padecido un cáncer, no encuentran en muchas ocasiones una respuesta terapéutica adecuada para las secuelas derivadas de los tratamientos del mismo. Los objetivos del presente estudio son: 1) Diseñar e implementar el sistema PaiNEd para la evaluación y el tratamiento basado en la Educación en Neurociencia del Dolor (END) en supervivientes cáncer con secuelas derivadas de los tratamientos médicos; 2) Comprobar la efectividad del sistema PaiNEd de forma integrada en un programa de recuperación física multimodal en comparación con la información biomédica tradicional. A este respecto, existe una escasez de propuestas para ciertos subgrupos de pacientes que demandan una especial atención. Este proyecto pretende llevar a cabo en primer lugar el diseño y puesta en marcha de un sistema de e-health para la evaluación y el tratamiento del dolor. En segundo lugar se realizará un estudio experimental aleatorizado controlado en el que se reclutarán 72 supervivientes de cáncer que serán asignados de forma aleatoria a los tres grupos de estudio: a) programa de recuperación física + acceso al sistema PaiNEd; b) programa de recuperación física + recomendaciones biomédicas tradicionales; c) grupo control. La evaluación se llevará a cabo de forma basal (al inicio del estudio), a las 8 semanas (momento de finalización de la intervención) y a los 6 meses de seguimiento de los pacientes.

2. PROYECTO DE INVESTIGACIÓN Y SUS RESULTADOS

2.1. ANTECEDENTES Y ESTADO ACTUAL DEL TEMA DE ESTUDIO

El cáncer sigue constituyendo una de las principales causas de morbi-mortalidad del mundo. De acuerdo con los últimos datos disponibles estimados dentro del proyecto GLOBOCAN, el número de tumores continúa creciendo, habiendo aumentado desde los 14 millones de casos en el mundo estimados en el año 2012 a los 18,1 millones en 2018 (Bray y cobs. 2018). Sin embargo, los procesos oncológicos destacan como entidades que poco a poco han ido aumentado su superviviencia, en la mayor parte de las localizaciones, convirtiéndose en procesos patológicos crónicos. El incremento de personas que superan un cáncer es sin duda una magnífica noticia propiciada por los avances en el diagnóstico y el tratamiento, pero al mismo tiempo suponen un incremento de carga de enfermedad a consecuencia de los efectos secundarios crónicos derivados del tratamiento oncológico (Alfano y cobs. 2013). La sociedad española de Oncología Médica estima que cada año habrá 100.000 nuevos supervivientes con necesidades especiales que necesitan ser definidas de forma adecuada. Los aspectos físicos sociales, laborales, psicológicos y emocionales de estos supervivientes tienen que estar caracterizados investigados y tratados por equipos multidisciplinares e interdisciplinares.

Las armas terapéuticas fundamentales en el tratamiento del cáncer son: la cirugía, la radioterapia y la quimioterapia, con el objetivo de maximizar la supervivencia mientras se preserva al máximo la forma física y la función (NCCN, 2016). La cirugía puede implicar la eliminación o alteración de ciertas estructuras anatómicas, lo que produce importantes efectos secundarios que afectan negativamente a calidad de vida de los pacientes (Rathod y cobs. 2015). Por otro lado, estas

técnicas quirúrgicas pueden ocasionar un deterioro en la función por pérdida de movilidad en algunas articulaciones como el hombro o el cuello en supervivientes de cáncer de mama (Shamley y cobs. 2012) o de cabeza y cuello (Teymoortash y cobs. 2010), afectación de estructuras nerviosas, así como alteraciones sensitivas, dolor (Agha-Mir-Salim y cobs. 2002) y pérdida de fuerza muscular (Cappiello y cobs. 2005). Además, los tratamientos con radio y quimioterapia pueden provocar problemas como mucositis, constricción (Pauloski, 2008) o dolor nociceptivo y neuropático (Wang y cobs. 2013; Binczak y cobs. 2014). A todo esto, habría que sumar otros efectos secundarios de los tratamientos, así como del propio cáncer: pérdida de peso, pérdida de masa muscular, la fatiga, etc. (Epstein y cobs. 2001; Couch y cobs. 2007). Algunos de los efectos de la terapia aguda pueden persistir, volviéndose permanentes o tardíos, desarrollándose hasta 90 días después del final del tratamiento (Ganzer y cobs. 2015), incluso en los primeros 5 años después del mismo (Chrischilles y cobs. 2019). Algunos autores consideran que más de un 60% de los pacientes tiene secuelas para las que no encuentra respuesta terapéutica (Ringash, 2015).

La alta tasa de supervivencia, en algunos casos es cercana al 90% a los 5 años (Benson y cobs. 2014) requiere con urgencia nuevas estrategias para abordar mejor las cuestiones de calidad de vida y satisfacer las necesidades de las personas que han sufrido un cáncer (Ringash, 2015). Existe una laguna de conocimiento sobre cuáles son estos déficits funcionales en algunas localizaciones, así como la cuantificación de estos déficits respecto a población general. Esta información nos permitiría implementar propuestas de recuperación física basadas en datos objetivos.

Estudios previos han contribuido a describir una hipersensibilidad muscular generalizada, como signo de un mecanismo central de sensibilización en supervivientes de ciertos tipos de cáncer como mama y colon (Fernández-Lao y cobs. 2010; Sánchez-Jiménez y cobs. 2014). El dolor podría atribuirse al daño de los nervios periféricos durante la cirugía (Cheville y cobs. 2009) o al efecto de la radioterapia pudiendo causar plexopatía braquial (Johansson y cobs. 2002). Además, ha sido descrita la fibrosis inducida por radiación, que conduce directamente a dolor neuropático, pérdida sensorial, espasmos musculares dolorosos, debilidad muscular, distonía cervical, contracturas, pérdida de elasticidad de los tejidos, osteoradionecrosis, adherencia de la piel a tejidos subyacentes (Hojan y cobs. 2014). Por último, la quimioterapia puede producir neuropatía periférica (Wolf y cobs. 2008) y aumentar el riesgo de daño del plexo braquial y con ello la sintomatología dolorosa del paciente. Recientemente nuestro de investigación, ha publicado un trabajo describiendo unos patrones de hipersensibilidad muscular parecidos a los descritos en las supervivientes de cáncer de mama (Ortiz-Comino y cobs. 2019). Todo lo expuesto origina un estado de sensibilización generalizada para el cuál la mayoría de los pacientes no encuentran solución terapéutica.

Además de la fatiga, el dolor es el síntoma más frecuente y persistente en después del cáncer y su tratamiento, debido, en gran medida a todo lo expuesto anteriormente. Se considera que el dolor interfiere con la capacidad física, emocional y laboral de los pacientes, perjudicando gravemente su calidad de vida y su participación en la sociedad. A este respecto, y teniendo en cuenta la naturaleza de los problemas, la terapia física se muestra como una opción de tratamiento adecuada para este tipo de pacientes. A pesar de no estar avalada por gran cantidad de estudios de calidad en el paciente oncológico, tiene el respaldo de resultados alentadores en muchas situaciones clínicas (Ajimsha, 2015). En la población de oncológica, un programa de terapia física individualizado se considera necesario para mejorar la calidad de vida en la fase de supervivencia, por lo que se considera recomendable un enfoque interdisciplinario temprano. La educación y el tratamiento oportuno pueden mejorar sustancialmente la calidad de vida de esta población.

En la búsqueda de protocolos de tratamiento más activos para las secuelas de los tratamientos del cáncer en general, existe evidencia científica que sugiere que, el control de ciertos componentes del estilo de vida como la actividad física y la nutrición es necesario en el proceso de supervivencia del mismo (Thomas y cobs. 2007). A este respecto, la actividad física se considera un tratamiento viable y efectivo para la mejora de la función física, la fuerza muscular, el manejo de la fatiga y calidad de

vida en los supervivientes de cáncer (Couch y cobs. 2007; Cantarero-Villanueva y cobs. 2012; Capozzi y cobs. 2016). Además, McNeely y cobs. (2008), mostraron una reducción significativa del dolor y la discapacidad en el hombro y mejora de la fuerza y la resistencia muscular de los mismos.

Por otro lado, en las últimas décadas la concienciación sobre el papel importante de las intervenciones educativas en el manejo del dolor en el cáncer ha aumentado (Bennet y cobs. 2009; Ling y cobs. 2012). Se ha demostrado que las intervenciones educativas generales son eficaces en la mejora de la severidad del dolor, la autoeficacia y el conocimiento y actitud ante el dolor en los pacientes de cáncer, sin embargo, los tamaños del efecto son moderados y tienen una relevancia clínica limitada. Esto puede ser explicado porque dichas intervenciones educativas se centran a menudo de forma fundamental en los tejidos y lesiones tisulares como fuente del dolor y restringidas generalmente al aspecto biomédico del mismo y asesoramiento general sobre actividad física y toma de analgésicos (Bennet y cobs. 2009; Ling y cobs. 2012).
El reciente avance en el conocimiento de sobre los mecanismos del dolor ha llevado a un enfoque más moderno, conocido como Educación en Neurociencia del Dolor (END) (Moseley & Butler 2015; Nijs y cobs. 2011; Nijs y cobs. 2014). Esta tendencia consiste en explicar la neurofisiología del dolor crónico y la capacidad del sistema nervioso de modular la experiencia del dolor, así como las influencias potenciales del sueño, los pensamientos y sentimientos, entre otros. La aplicación de la END podría mejorar la eficacia de las modalidades de terapia física actualmente aplicadas para la prevención y el tratamiento del dolor y las discapacidades relacionadas con el tratamiento del cáncer en comparación con una intervención biomédica tradicional. A este respecto, algunas experiencias de este tipo se están comenzando a poner en marcha en población oncológica de forma aislada en supervivientes de cáncer de mama (De Groef y cobs. 2018).

La necesidad de promover intervenciones individualizadas exige una aproximación urgente desde la investigación que pueda ajustarse a las necesidades de los pacientes de una manera costo-efectiva (Hudis & Jones, 2014). Para asegurar esta individualización en el tratamiento puede ser positivo contar con herramientas tecnológicas que aseguren la adherencia del paciente a ciertas recomendaciones. Esta situación se hace aún más necesaria en situaciones cómo la actual pandemia provocada por la COVID-19, en la que las intervenciones basadas en e-Health o Telerehabilitación supondrían la respuesta para muchas poblaciones en riesgo que no pudieran acceder a servicios presenciales por los riesgos que implicaría para su salud (Wyper y cobs 2020). Según nuestros datos no existen experiencias previas basadas en tecnología móvil que evalúen y emitan recomendaciones para dar soporte en la mejora del dolor en la población oncológica.

El grupo de investigación que lidera esta propuesta ha participado en diferentes proyectos previos que han acercado el mundo de las denominadas Tecnologías de la Información y la Comunicación (TICS) a la monitorización e implementación de recomendaciones terapéuticas en problemas de salud de elevada prevalencia y coste personal-económico en nuestra sociedad como el dolor de espalda (Palacín-Marín y cobs. 2013; Esteban-Moreno y cobs. 2014), los síntomas derivados del cáncer como la fatiga o el dolor (Galiano-Castillo y cobs. 2014a, 2014b), las fracturas de cadera (Ortiz Piña y cobs. 2019) o la implantación de sistemas de recomendaciones sobre equilibrio energético (ejercicio y nutrición) (Lozano-Lozano y cobs. 2019) con un éxito prometedor, tanto a nivel de científico como de impacto social (www.youtube.com/watch?v=j6A0nGRNFx8; www.cuidateconnosotros.com). Aunque la American Cancer Society (Cohen y cobs. 2016) recomienda la derivación de los pacientes de oncológicos con dolor, disfunción del hombro, neuropatía o trismus a los especialistas en rehabilitación, la evidencia científica al respecto es escasa, y en algunos casos prácticamente nula. Por lo tanto, serían necesarios trabajos de buena calidad metodológica que desarrollen estrategias de tratamiento específicas y efectivas para esta población de pacientes.

Además, para optimizar los recursos sanitarios es necesario conocer si las propuestas multimodales muestran la misma eficacia que la aplicación de un solo recurso terapéutico (como la terapia manual), esto permitirá simplificar el tratamiento a nivel clínico reduciendo el coste sanitario y el tiempo de tratamiento. Diferentes trabajos realizados por nuestro grupo de investigación han mostrado la eficacia de programas de rehabilitación multimodal (ejercicio terapéutico, cinesiterapia) en el abordaje de problemas relacionados con el dolor y la disfunción asociados al tratamiento

médico del cáncer de mama (Cantarero-Villanueva y cobs. 2012; Cantarero-Villanueva y cobs. 2013; Fernández-Lao y cobs. 2012b) o de colon (Cantarero-Villanueva y cobs, 2017). Consideramos, por tanto, que es posible que resultados similares puedan ser alcanzados con programas de recuperación física multimodal basado en las propuestas descritas en supervivientes de cáncer que presenten dolor. Para ello es necesario diseñar e implementar programas de tratamiento adecuados para esta población concreta.

Este proyecto pretende dar respuesta a través de la relación investigación-asistencia sanitaria, a una necesidad global de los supervivientes de cáncer, implementando estrategias que mejoren su calidad de vida y que a largo plazo puedan tener un impacto en su supervivencia.

2.2. BIBLIOGRAFÍA

- Agha-Mir-Salim P, Schulte-Mattler W, Funk U, Lautenschlager C, Bloching M, Berghaus A. Origin of shoulder pain after neck dissection^. Importance of the cervical plexus. HNO. 2002;50(6): 544–52.
- Ajimsha MS, Al-Mudahka NR, Al-Madzhar JA. Effectiveness of myofascial release: systematic review of randomized controlled trials. J Bodyw Mov Ther. 2015 Jan;19(1):102-12.
- American Cancer Society. Cancer Facts & Figures 2018. Atlanta: American Cancer Society; 2018. Disponible en: https://www.cancer.org/content/dam/cancer-org/research/cancer-facts-and-statistics/annual-cancer-facts-and-figures/2018/cancer-facts-and-figures-2018.pdf
- Bennett MI, Bagnall AM, José Closs S. How effective are patient based educational interventions in the management of cancer pain? Systematic review and meta-analysis. *Pain* 2009;143:192–9.
- Benson E, Li R, Eisele D, Fakhry C. The clinical impact of HPV tumor status upon head and neck squamous cell carcinomas. Oral Oncol. 2014;50:565-574.
- Binczak M, Navez M, Perrichon C, Blanchard D, Bollet M, Calmels P, Couturaud C, Dreyer C, Espitalier F, Testelin S, Albert S, Morinière S; SFORL Work Group. Management of somatic pain induced by head-and-neck cancer treatment: definition and assessment. Guidelines of the French Oto-Rhino-Laryngology- Head and Neck Surgery Society (SFORL). Eur Ann Otorhinolaryngol Head Neck Dis. 2014 Sep;131(4):243-7.
- Bray F, Ferlay J, Soerjomataram I, Siegel RL, Torre LA, Jemal A. Global Cancer Statistics 2018: GLOBOCAN Estimates of Incidence and Mortality Worldwide for 36 Cancers in 185 Countries. CA Cancer J Clin. 2018 [Disponble en: https://onlinelibrary.wiley.com/doi/full/10.3322/caac.21492.]
- Cantarero-Villanueva I, Cuesta-Vargas AI, Lozano-Lozano M, Fernández-Lao C, Fernández-Pérez A, Galiano-Castillo N. Changes in Pain and Muscle Architecture in Colon Cancer Survivors After a Lumbopelvic Exercise Program: A Secondary Analysis of a Randomized Controlled Trial. Pain Med. 2017 Jul 1;18(7):1366-1376.
- Cantarero-Villanueva I, Fernández-Lao C, Caro-Morán E, Morillas-Ruiz J, Galiano- astillo N, Díaz-Rodríguez L, Arroyo-Morales M. Aquatic exercise in a chest-high pool for hormone therapy-induced arthralgia in breast cancer survivors: a pragmatic controlled trial. Clin Rehabil. 2013 Feb;27(2):123-32.
- Cantarero-Villanueva I, Fernández-Lao C, Fernández-de-Las-Peñas C, López-Barajas IB, Del-Moral-Ávila R, de la-Llave-Rincón AI, Arroyo-Morales M. Effectiveness of water physical therapy on pain, pressure pain sensitivity, and myofascial trigger points in breast cancer survivors: a randomized, controlled clinical trial. Pain Med. 2012 Nov;13(11):1509-19.
- Cappiello J, Piazza C, Giudice M, De Maria G, Nicolai P. Shoulder disability after different selective neck dissections (levels II–IV versus levels II–V): a comparative study. Laryngoscope. 2005;115(2):259–63.
- Cheville AL, Tchou J. Barriers to rehabilitation following surgery for primary breast cancer. J SurgOncol 2007;95(5):409–418.
- Chrischilles EA, Riley D, Letuchy E, et al. Upper extremity disability and quality of life after breast cancer treatment in the Greater Plains Collaborative clinical research network. Breast Cancer Res Treat. 2019;175(3):675-689.
- Couch M, Lai V, Cannon T, Guttridge D, Zanation A, George J, Hayes DN, Zeisel S, Shores C. Cancer cachexia syndrome in head and neck cancer patients: part I.Diagnosis, impact on quality of life and survival, and treatment. Head Neck. 2007 Apr;29(4):401-11.

- De Groef A, Devoogdt N, Van der Gucht E, *et al*. EduCan trial: study protocol for a randomised controlled trial on the effectiveness of pain neuroscience education after breast cancer surgery on pain,physical, emotional and workrelated functioning. *BMJ Open* 2019;9:e025742.
- Epstein JB, Robertson M, Emerton S, Phillips N, Stevenson-Moore P. Quality of life and oral function in patients treated with radiation therapy for head and neck cancer. Head Neck. 2001 May;23(5):389-98.
- Fernández-Lao C, Cantarero-Villanueva I, Fernández-de-Las-Peñas C, del Moral-Ávila R, Castro-Sánchez AM, Arroyo-Morales M. Effectiveness of a multidimensional physical therapy program on pain, pressure hypersensitivity, and trigger points in breast cancer survivors: a randomized controlled clinical trial. Clin J Pain. 2012 Feb;28(2):113-21.
- Hojan K, Milecki P. Opportunities for rehabilitation of patients with radiation fibrosis syndrome. Reports of Practical Oncology and Radiotherapy. 2014;19:1-6.
- Johansson S, Svensson H, Denekamp J. Dose response and latency for radiation-induced fibrosis, edema, and neuropathy in breast cancer patients. Int J RadiatOncolBiolPhys 2002;52(5):1207–1219.
- Ling CC, Lui LY, So WK. Do educational interventions improve cancer patients' quality of life and reduce pain intensity? Quantitative systematic review. *J Adv Nurs* 2012;68:511–20.
- Lozano-Lozano M, Cantarero-Villanueva I, Martin-Martin L, Galiano-Castillo N, Sanchez MJ, Fernández-Lao C, Postigo-Martin P, Arroyo-Morales M. A Mobile System to Improve Quality of Life Via Energy Balance in Breast Cancer Survivors (BENECA mHealth): Prospective Test-Retest Quasiexperimental Feasibility Study. *JMIR Mhealth Uhealth*. 2019;7(6):e14136.
- Moseley GL, Butler DS. Fifteen years of explaining pain: the past, present, and future. *J Pain* 2015;16:807–13.
- National Comphrehensive Cancer Network. Head and neck cancers, version 1; 2016. Disponible en: https://www.nccn.org/professionals/ physician_gls/PDF/head-and-neck.pdf.
- Nijs J, Paul van Wilgen C, Van Oosterwijck J, *et al*. How to explain central sensitization to patients with 'unexplained' chronic musculoskeletal pain: practice guidelines. *Man Ther* 2011;16:413–8.
- Nijs J, Torres-Cueco R, van Wilgen CP, *et al*. Applying modern pain neuroscience in clinical practice: criteria for the classification of central sensitization pain. *Pain Physician* 2014;17:447–57.
- Ortiz-Comino L, Fernández-Lao C, Castro-Martín E, et al. Myofascial pain, widespread pressure hypersensitivity, and hyperalgesia in the face, neck, and shoulder regions, in survivors of head and neck cancer. *Support Care Cancer*. 2020;28(6):2891-2898.
- Ortiz-Piña M, Salas-Fariña Z, Mora-Traverso M, Martín-Martín L, Galiano-Castillo N, GarcíaMontes I, Cantarero-Villanueva I, Fernández-Lao C, Arroyo-Morales M, Mesa-Ruíz A, CastelloteCaballero Y, Salazar-Graván S, Kronborg L, Martín-Matillas M, Ariza-Vega P. A home-based telerehabilitation protocol for patients with hip fracture called @ctivehip. Res Nurs Heal. 2019
- Pauloski BR. Rehabilitation of dysphagia following head and neck cancer. Phys Med Rehabil Clin N Am. 2008;19(4):889-928).
- Rathod S, Livergant J, Klein J,Witterick I, Ringash J. A systematic review of quality of life in head and neck cancer treated with surgery with or without adjuvant treatment. Oral Oncol. 2015;51(10): 888–900.
- Sánchez-Jiménez A, Cantarero-Villanueva I, Molina-Barea R, Fernández-Lao C, Galiano-Castillo N, Arroyo-Morales M. Widespread pressure pain hypersensitivity and ultrasound imaging evaluation of abdominal area after colon cancer treatment. Pain Med. 2014 Feb;15(2):233-40.
- Shamley D, Lascurain-Aguirrebeña I, Oskrochi R, Srinaganathan R. Shoulder morbidity after treatment for breast cancer is bilateral and greater after mastectomy. Acta Oncol. 2012;51(8):1045-1053.
- Wyper GMA, Assunção R, Cuschieri S, Devleeschauwer B, Fletcher E, Haagsma JA, et al. Population vulnerability to COVID-19 in Europe: a burden of disease analysis. Arch Public Health. 2020;78:47.

2.3. HIPÓTESIS, PREGUNTA DE INVESTIGACIÓN O ESTUDIO DESCRIPTIVO

La hipótesis del presente proyecto es que tanto un programa de recuperación física (ejercicio terapéutico y técnicas de recuperación miofascial) en combinación con el uso de la app de educación, como un programa de recuperación física (ejercicio terapéutico y técnicas de recuperación miofascial) en combinación con las recomendaciones biomédicas habituales serán efectivos en el tratamiento del dolor y la disfunción de los

pacientes de cáncer tras los tratamientos médicos, mejorando de esta forma la calidad de vida de los mismos. El programa de recuperación física en combinación con el uso de PainEd representará una mejora más importante en esta población. Esta mejoría se mantendrá tras un período de seguimiento.

PLANTEAMIENTO DEL PROBLEMA.

Los beneficios de aplicación de los programas multimodales como forma de recuperación en población emergente que supera un cáncer están mostrando una evidencia científica suficiente que justifica la aparición de nuevos servicios de salud que den respuesta a las necesidades de estos pacientes durante el proceso de recuperación. En este sentido:

a) la mayor incidencia de la enfermedad y el éxito terapéutico debido al diagnóstico precoz incrementan el número de personas que superan el cáncer, si bien los efectos secundarios derivados del tratamiento deben ser atendidos por el sistema sanitario.

b) no existen programas de rehabilitación que hayan añadido la Educación en Neurociencia del Dolor dentro de las propuestas terapéuticas estandarizadas y útiles para este grupo poblacional.

c) no existe información sobre como este tipo de servicios de rehabilitación pueden reducir los trastornos músculoesqueléticos en este tipo de pacientes y mejorar el resto de secuelas físicas, y en consecuencia la calidad de vida de los mismos.

2.4. OBJETIVOS

OE1: Diseñar y definir el sistema de evaluación PaiNEd.
En este primer OE a través de la “expertise” de los diferentes agregados se definirán los contenidos específicos de evaluación del dolor y la discapacidad física para que se adapten con la mayor precisión a la población diana de este estudio.

OE2: Diseñar y definir el sistema de educación PaiNEd
Implementación de sistema de recomendaciones basado en la evidencia actualmente disponible sobre la educación en neurociencia del dolor y en los resultados obtenidos en la fase de monitorización del sistema.

OE3: Desarrollar la tecnología y arquitectura técnica del sistema PaiNEd
Desarrollo tecnológico del sistema en su versión inicial, puesta a punto del sistema para su utilización en situaciones clínicas reales. Fase de monitorización interna del sistema.

OE4. Ensayo clínico aleatorizado controlado. Estudio de efectividad
El objetivo será examinar la efectividad de una herramienta sobre END como suplemento de un programa de tratamiento multimodal basado en ejercicio terapéutico y técnicas de recuperación miofascial, para tratar el dolor y la disfunción en supervivientes de cáncer en comparación con un programa de tratamiento multimodal combinado con la información biomédica tradicional.

2.5. METODOLOGÍA

Los participantes serán pacientes con cáncer mama que hayan finalizado el tratamiento y hasta dos años tras haberlo finalizado. La muestra será reclutada desde los servicios de Oncología Médica y/o Oncología Radioterápica del Hospital Virgen de las Nieves y el Hospital de Parque Tecnológico de la Salud de Granada.

Criterios de inclusión: a) Sujetos mayores de 18 años. b) Haber sido intervenido con cirugía y terminado tratamiento coadyuvante (radioterapia y/o quimioterapia), desde hace seis meses a dos años, c) No tener cáncer activo, d) Tener dolor en las regiones relacionadas con la zona del tumor (cervical y/o braquial y/u hombro), dolor ≥ 4 (VAS 0 a 10) durante >4 semanas e) Presentar alteraciones musculoesqueléticas o funcionales en las anteriores regiones.

Criterios de exclusión: a) Identificación por el equipo de investigación de imposibilidad física o mental para realizar las pruebas y test del estudio, b) Padecer dolor crónico o haber sufrido un

traumatismo anterior en la columna vertebral, la cabeza, Articulación Témporomandibular o las extremidades superiores.

Diseño estudio: Diseño experimental con dos grupos de intervención y un grupo control. Estudio de efectividad. Los criterios de inclusión y exclusión serán los mismos que para la fase anterior.

GE1: programa de rehabilitación multimodal + END

GE2: programa de rehabilitación multimodal + información biomédica tradicional

GC: grupo control (Lista de espera de pacientes)

Cálculo del tamaño muestral. La estimación del tamaño de la muestra y potencia para el ESTUDIO II se han determinado para la variable principal, el dolor medido a través de la Escala Visual Analógica (EVA) (Jensen, 1993). Basándonos en un estudio previo (De Groef y cobs. 2017) y estimando que los pacientes en el grupo de intervención tendrían una diferencia de 15mm o más en la EVA en comparación con el grupo control, se podrán detectar diferencias de al menos un 5% con una potencia del 80% y un nivel de alfa α 0,05. Para ello será necesaria una muestra de 28 pacientes por grupo, que dará un total de 72 supervivientes de CCC, lo que permitirá una pérdida máxima de seguimiento del 10%. Para realizar este cálculo muestral se utilizó el Software Epidat 4.2.

Aleatorización y cegamiento: Los participantes que cumplan con los criterios de inclusión serán aleatorizados en cada uno de los grupos utilizando un programa de generación de números aleatorios (EPIDAT 4.2, Xunta de Galicia). La secuencia se introducirá en un sobre opaco cerrado por un miembro externo a la investigación y serán abiertos una vez completada la valoración basal, por lo que el personal de valoración estará enmascarado en cuanto a la aleatorización de las participantes, reduciendo así el riesgo de sesgos durante la valoración. El cegamiento de evaluadores estará garantizado por hecho de que algunos integrantes del grupo están especializados en el tratamiento del paciente oncológico y otros en la evaluación de los mismos.

INTERVENCIONES

GRUPO EXPERIMENTAL 1: PROGRAMA DE REHABILITACIÓN MULTIMODAL (EJERCICIO TERAPEUTICO + TERAPIA MANUAL). Recibirá la intervención a través de la participación en el programa de recuperación física (multimodal) con una duración de 8 semanas. Los pacientes recibirán tres sesiones semanales en días alternos con una duración de 60 min. Las sesiones consistirán en trabajo grupal de ejercicio terapéutico: a) calentamiento (10 min trabajo aeróbico), b) movilizaciones activas (cervical/hombro) c) trabajo de fuerza de la musculatura cervical y del hombro + trabajo de acondicionamiento general d) trabajo de control postural e) estiramientos, relajación. Además, los pacientes recibirán un total de 4 sesiones de terapia manual individual (1 sesión cada 2 semas). Por último, los pacientes tendrán acceso al sistema PaiNEd antes del comienzo del programa (para prepararlos para las sesiones de tratamiento) y durante el desarrollo del programa de tratamiento en el que introducirán los niveles y características de dolor y recibirán una serie de consejos educativos y recomendaciones para la mejora del mismo.

GRUPO EXPERIMENTAL 2: PROGRAMA DE TRATAMIENTO MEDIANTE TERAPIA MANUAL. Recibirá la intervención a través de la participación en el programa de recuperación física (multimodal) con una duración de 8 semanas. Los pacientes recibirán tres sesiones semanales en días alternos con una duración de 60 min. El programa consistirá en el mismo tratamiento propuesto para el GE1 pero los pacientes recibirán un dosier con las recomendaciones biomédicas tradicionales sobre el manejo del dolor y la discapacidad asociada a los efectos secundarios del tratamiento por el cáncer.

GRUPO CONTROL. Lista de espera de pacientes que hayan aceptado participar en el estudio. Recibirán un dossier de información con recomendaciones sobre el control del dolor y mejora de la

disfunción (similar a lo trabajado en el grupo experimental). Dichas recomendaciones serán repartidas al inicio del estudio (valoración inicial) y se les citará a las 8 semanas para la siguiente toma de variables. Todos los pacientes serán invitados a participar el en la fase experimental al finalizar la fase de control.

VARIABLES PARA EL ENSAYO CLÍNICO ALEATORIZADO CONTROLADO (OE4)
Las variables de estudio serán evaluadas en condiciones basales, al finalizar la intervención de 2 meses y 6 meses tras la finalización de la misma.

Se suministrará un cuestionario de variables sociodemográficas al inicio de la valoración. Los datos clínicos se tomarán de la historia electrónica por parte del personal sanitario participante.

Variables de dolor y función

Dolor: Escala Visual Analógica para valorar dolor de cuello, zona temporomandibular y hombro (Fernández-Lao y cobs. 2010) y Breve Inventario del Dolor (para valorar cualidad, localización e interferencia del dolor. Galiano-Castillo y cobs. 2014)

Inventario de Sensibilización Central. Presencia y grado de hipersensibilidad a los estímulos no mecánicos (Mayer y cobs. 2012).

Umbrales de dolor a la presión (Algometría): Se realizará en los puntos relacionados con la zona cervical y de la cintura escapular (articulación C5-C6, trapecio superior, elevador de la escápula, masetero, deltoides, articulación esternoclavicular y el punto a distancia del tibial anterior). (Fernández-Lao y cobs. 2010; 2011).

Catastrofización relacionalda con el dolor: Escala de Catastrofización del Dolor: Utilizado para valorar el pensamiento catastrófico relacionado con el dolor (Sullivan y cobs. 1995).

Goniometría activa hombro y columna cervical: Mediante la utilización de goniómetro e inclinómetro cervical. (Cantarero-Villanueva y cobs. 2011)

Variables de Calidad de Vida y condición física

Calidad de vida: Cuestionarios general de cáncer y específico (EORT QLQ-C30 + módulo mama [BR-23]) (Singer y cobs. 2009).

Fatiga: Cuestionario de Fatiga de Piper (Cantarero-Villanueva y cobs. 2014)

Capacidad funcional: A través del test de 6 minutos marcha (Laskin y cobs. 2007).

Fuerza de prensión manual: Mediante dinamometría isométrica manual (España-Romero, 2010).

Evaluación fuerza resistencia musculatura profunda cervical: Mediante test de flexión cráneo-cervical y ecografía musculoesquética y ecografía musculoesquelética (Edmonston y cobs. 2008)

Composición corporal: Mediante un análisis de bioimpedancia eléctrica (Inbody 720) (Galiano-Castillo y cobs 2014).

Variabilidad de la frecuencia cardíaca: A través de una grabadora holter (Fernández-Lao y cobs. 2011).
Respuesta inflamatoria y respuesta de estrés: Biomarcadores salivales de Cortisol, IL6 y PCR. Esta determinación se realizará para valorar el efecto del programa de recuperación física frente a las recomendaciones del grupo control siguiendo procedimientos estandarizados en el laboratorio de la Facultad de Ciencias de la Salud. (Cantarero-Villanueva y cobs. 2012).

Análisis estadístico

Para analizar los resultados se usará el programa estadístico IBM SPSS 24.0. El Test de Kolmogorov-Smirnov será aplicado para comprobar la distribución normal de las variables ($P>0.05$). Se incluirán curvas ROC para determinar la exactitud de los tests. La estadística descriptiva de este estudio utilizará valores de media y desviación estándar junto con intervalos de confianza al 95%. Los valores previos de cada variable de seguimiento serán comparados a través del test de la T-Student para muestras independientes. Desde el punto de vista de la estadística inferencial y para dar respuesta al objetivo principal de este estudio se realizará un análisis por intención de tratar. Se llevará a cabo un modelo de medidas repetidas de ANOVA 3x3 utilizando como factor intra-sujeto el tiempo (basal, post-intervención, seguimiento) y como factor intersujeto la intervención (GE1, GE2, control). El test de Bonferroni será utilizado para el análisis post-hoc. Para el estudio sobre subgrupos con posibles resultados diferenciales (estadios elevados tumor, edad, género) serán utilizadas como posibles variables confusoras en un análisis de regresión múltiple posterior. Se considerará un nivel de significación estadístico de p-valor menor a 0,05.

2.6. PLAN DE TRABAJO

Año 1º Actividad	1 Mes	2 Mes	3 Mes	4 Mes	5 Mes	6 Mes	7 Mes	8 Mes	9 Mes	10 Mes	11 Mes	12 Mes
PT1												
PT2												
PT3												
Año 2º Actividad	13 Mes	14 Mes	15 Mes	16 Mes	17 Mes	18 Mes	19 Mes	20 Mes	21 Mes	22 Mes	23 Mes	24 Mes
PT3												
PT4												
Año 3º Actividad	25 Mes	26 Mes	27 Mes	28 Mes	29 Mes	30 Mes	31 Mes	32 Mes	33 Mes	34 Mes	35 Mes	36 Mes
PT4												
PT5												
PT6												

TITLE OF THE PROJECT

EFFECTIVENESS OF AN E-HEALTH SYSTEM INTEGRATED IN A PHYSICAL RECOVERY PROGRAM FOR THE TREATMENT OF PAIN IN CANCER SURVIVORS. PaiNEd STUDY

KEYWORDS

Cancer, pain, sequelae, education, multimodal rehabilitation

3. ABSTRACT

People who have suffered from cancer often do not find an adequate therapeutic response for the sequelae derived from its treatment. The objectives of this study are: 1) To design and implement the PaiNEd system for the evaluation and treatment based on Pain Neuroscience Education (PNE) in cancer survivors with sequelae derived from medical treatments. 2) To test the effectiveness of the PaiNEd system in a multimodal physical recovery program compared to traditional biomedical information. In this regard, there is a shortage of proposals for certain subgroups of patients who require special attention. First of all, this project intends to carry out the design and implementation of an e-health system for the evaluation and treatment of pain. Secondly, a randomized controlled trial study will be carried out in which 72 cancer survivors will be recruited and randomly assigned to the three study groups: a) physical recovery program + access to the PaiNEd system; b) physical recovery program + traditional biomedical recommendations; c) control group. Assessments will be carried out at baseline (at the beginning of the study), at 8 weeks (time of completion of the intervention) and at 6 months of follow-up of the patients.

4. RESEARCH PROJECT AND ITS RESULTS

4.1. BACKGROUND AND CURRENT STATUS OF THE SUBJECT OF STUDY

Cancer continues to be one of the leading causes of morbidity and mortality in the world. According to the latest available data estimated within the GLOBOCAN project, the number of tumors continues to grow, having increased from the estimated 14 million cases in the world in 2012 to 18.1 million in 2018. (Bray et al. 2018). However, oncological processes stand out as entities that have gradually increased their survival, becoming chronic pathological processes in most cancer locations. The increase in people who overcome cancer is undoubtedly great news fostered by advances in diagnosis and treatment, but at the same time they represent an increase in the burden of disease as a result of the chronic side effects derived from cancer treatment. (Alfano et al. 2013). The Spanish Society of Medical Oncology estimates that each year there will be 100,000 new survivors with special needs that need to be adequately adressed. The physical, social, psychological and emotional aspects of these survivors have to be characterized, investigated and treated by multidisciplinary and interdisciplinary teams.

The main therapeutic agents in the treatment of cancer are: surgery, radiotherapy and chemotherapy, with the aim of maximizing survival while preserving physical fitness and function as much as possible (NCCN, 2016). Surgery may involve the removal or alteration of certain anatomical structures, which produces significant side effects that negatively affect the quality of life of patients. (Rathod et al. 2015). On the other hand, these surgical procedures can cause impaired function due to loss of mobility in some joints such as shoulder or neck in survivors of breast cancer (Shamley et al. 2012), involvement of nervous structures, as well as sensory alterations, pain (Agha-Mir-Salim et al. 2002) and loss of muscle strength (Cappiello et al. 2005). In addition, radiotherapy and chemotherapy treatments can cause mucositis, stricture (Pauloski, 2008), or nociceptive and neuropathic pain (Wang et al. 2013; Binczak et al. 2014). To all this, we should add other side effects of the treatments, as well as of the cancer itself: weight loss, loss of muscle mass, fatigue, etc. (Epstein et al. 2001; Couch et al. 2007). Some of the effects of acute therapy may persist, becoming permanent or delayed, developing up to 90 days after the end of treatment (Ganzer et al. 2015), even in the first 5 years after treatment (Chrischilles et al. 2019). Some authors consider that more than 60% of patients have sequelae for which they find no therapeutic response (Ringash, 2015).

The high survival rate, in some cases close to 90% at 5 years (Benson et al. 2014) urgently requires new strategies to better address quality of life issues and meet the needs of people who have suffered cancer (Ringash, 2015). There is a knowledge gap regarding what these functional deficits are in some locations, as well as the quantification of these deficits with respect to the general population. This information would allow us to implement physical recovery proposals based on objective data.

Previous studies have contributed to describe generalized muscle hypersensitivity, as a sign of a central sensitization mechanism in survivors of certain types of cancer such as breast and colon

(Fernández-Lao et al. 2010; Sánchez-Jiménez et al. 2014). Pain could be attributed to peripheral nerve damage during surgery (Cheville et al. 2009) or to the effect of radiotherapy which may cause brachial plexopathy (Johansson et al. 2002). In addition, radiation-induced fibrosis has been described, leading directly to neuropathic pain, sensory loss, painful muscle spasms, muscle weakness, cervical dystonia, contractures, loss of tissue elasticity, osteoradionecrosis, skin adherence to underlying tissues (Hojan et al. 2014). Finally, chemotherapy can cause peripheral neuropathy (Wolf et al. 2008) and increase the risk of damage to the brachial plexus and with it the patient's painful symptoms. Our research group has recently published a study describing patterns of muscle hypersensitivity similar to those described in breast cancer survivors (Ortiz-Comino et al. 2019). All this, causes a state of generalized sensitization for which the majority of patients do not find a therapeutic solution.

In addition to fatigue, pain is the most frequent and persistent symptom after cancer and its treatment, largely due to all of the above. Pain is considered to interfere with the physical, emotional and work capacity of patients, seriously impairing their quality of life and their participation in society. In this regard, and taking into account the nature of the problems, physical therapy appears to be an appropriate treatment option for this type of patient. Despite not being supported by a large number of quality studies in cancer patients, it is supported by encouraging results in many clinical situations (Ajimsha, 2015). In the oncological population, an individualized physical therapy program is considered necessary to improve quality of life in the survival phase, so an early interdisciplinary approach is considered advisable. Education and timely treatment can substantially improve the quality of life of this population.

In the search for more active treatment protocols for the sequelae of cancer treatments in general, there is scientific evidence that suggests that monitoring certain lifestyle components such as physical activity and nutrition is necessary in the survival process (Thomas et al. 2007). In this regard, physical activity is considered a viable and effective treatment for improving physical function, muscle strength, fatigue management, and quality of life in cancer survivors (Couch et al. 2007; Cantarero-Villanueva et al. 2012; Capozzi et al. 2016). Also, McNeely et al. (2008), showing a significant reduction in shoulder pain and disability and improvement in shoulder strength and muscular endurance.

On the other hand, awareness in recent decades of the important role of educational interventions in cancer pain management has increased (Bennet et al. 2009; Ling et al. 2012). General educational interventions have been shown to be effective in improving pain severity, self-efficacy, and pain knowledge and attitudes in cancer patients, however, effect sizes are modest and of limited clinical relevance. This can be explained because such educational interventions often focus mainly on tissue lesions as the source of pain and are generally restricted to the traditional biomedical aspect of pain and general advice on physical activity and taking analgesics (Bennett et al. 2009; Ling et al. 2012). Recent advances in knowledge of pain mechanisms have led to a more modern approach, known as Pain Neuroscience Education (PNE) (Moseley & Butler 2015; Nijs et al. 2011; Nijs et al. 2014). This trend, aims to explain the neurophysiology of chronic pain and the ability of the nervous system to modulate the experience of pain, as well as the potential influences of sleep, thoughts and feelings, among others. The application of PNE could improve the efficacy of currently applied physical therapy modalities for the prevention and treatment of pain and disabilities related to cancer treatment compared to traditional biomedical intervention. In this regard, some experiences of this type are beginning to be implemented in the cancer population in breast cancer survivors (De Groef et al. 2018).

The need to promote individualized interventions requires an urgent approach from research that can be adjusted to the needs of patients in a cost-effective way (Hudis & Jones, 2014). To ensure this individualization in treatment, it may be positive to have technological tools that ensure patient adherence to certain recommendations. This situation becomes even more necessary in situations such as the current pandemic caused by COVID-19, in which interventions based on e-Health or Telerehabilitation would be the answer for many populations at risk who could not access face-to-face services due to the risks that could imply for their health (Wyper et al. 2020). According to our

data, there are no previous experiences based on mobile technology that evaluate and give recommendations to support the improvement of pain in the cancer population.

The research group leading this proposal has participated in different previous projects that have brought the world of Information and Communication Technologies (ICTs) closer to monitoring and implementing therapeutic recommendations in health problems with high prevalence and personal cost-economic in our society such as back pain (Palacín-Marín et al. 2013; Esteban-Moreno et al. 2014), symptoms derived from cancer such as fatigue or pain (Galiano-Castillo et al. 2014a, 2014b), hip fractures (Ortiz Piña et al. 2019) or the implementation of systems of recommendations on energy balance (exercise and nutrition) (Lozano-Lozano et al. 2019) with promising success, both at the scientific and social impact aspect. (www.youtube.com/watch?v=j6A0nGRNFx8; www.cuidateconnosotros.com). Although the American Cancer Society (Cohen et al. 2016) recommends the referral of cancer patients with pain, shoulder dysfunction, neuropathy, or trismus to rehabilitation specialists, scientific evidence in this regard is scarce, and in some cases practically non-existent. Therefore, works of good methodological quality would be necessary to develop specific and effective treatment strategies for this population of patients.

In addition, in order to optimize health resources, it is necessary to know if multimodal proposals show the same efficacy as the application of a single therapeutic resource (such as manual therapy). This will simplify treatment at the clinical level, reducing health costs and treatment time. Different works carried out by our research group have shown the efficacy of multimodal rehabilitation programs (therapeutic exercise, kinesitherapy) in addressing problems related to pain and dysfunction associated with the medical treatment of breast cancer (Cantarero-Villanueva et al. 2012; Cantarero-Villanueva et al. 2013; Fernández-Lao et al. 2012b) or colon (Cantarero-Villanueva et al. 2017). Therefore, we believe that it is possible that similar results could be achieved with multimodal physical recovery programs based on the proposals described in cancer survivors with pain. For this, it is necessary to design and implement treatment programs suitable for this specific population.

This project aims to respond through the research-health care relationship, to a global need of cancer survivors, implementing strategies that improve their quality of life and that in the long term can have an impact on their survival.

4.2. REFERENCES

- Agha-Mir-Salim P, Schulte-Mattler W, Funk U, Lautenschlager C, Bloching M, Berghaus A. Origin of shoulder pain after neck dissection^. Importance of the cervical plexus. HNO. 2002;50(6): 544–52.
- Ajimsha MS, Al-Mudahka NR, Al-Madzhar JA. Effectiveness of myofascial release: systematic review of randomized controlled trials. J Bodyw Mov Ther. 2015 Jan;19(1):102-12.
- American Cancer Society. Cancer Facts & Figures 2018. Atlanta: American Cancer Society; 2018. Disponible en: https://www.cancer.org/content/dam/cancer-org/research/cancer-facts-and-statistics/annual-cancer-facts-and-figures/2018/cancer-facts-and-figures-2018.pdf
- Bennett MI, Bagnall AM, José Closs S. How effective are patient based educational interventions in the management of cancer pain? Systematic review and meta-analysis. *Pain* 2009;143:192–9.
- Benson E, Li R, Eisele D, Fakhry C. The clinical impact of HPV tumor status upon head and neck squamous cell carcinomas. Oral Oncol. 2014;50:565-574.
- Binczak M, Navez M, Perrichon C, Blanchard D, Bollet M, Calmels P, Couturaud C, Dreyer C, Espitalier F, Testelin S, Albert S, Morinière S; SFORL Work Group. Management of somatic pain induced by head-and-neck cancer

treatment: definition and assessment. Guidelines of the French Oto-Rhino-Laryngology- Head and Neck Surgery Society (SFORL). Eur Ann Otorhinolaryngol Head Neck Dis. 2014 Sep;131(4):243-7.

- Bray F, Ferlay J, Soerjomataram I, Siegel RL, Torre LA, Jemal A. Global Cancer Statistics 2018: GLOBOCAN Estimates of Incidence and Mortality Worldwide for 36 Cancers in 185 Countries. CA Cancer J Clin. 2018 [Disponble en: https://onlinelibrary.wiley.com/doi/full/10.3322/caac.21492.]
- Cantarero-Villanueva I, Cuesta-Vargas AI, Lozano-Lozano M, Fernández-Lao C, Fernández-Pérez A, Galiano-Castillo N. Changes in Pain and Muscle Architecture in Colon Cancer Survivors After a Lumbopelvic Exercise Program: A Secondary Analysis of a Randomized Controlled Trial. Pain Med. 2017 Jul 1;18(7):1366-1376.
- Cantarero-Villanueva I, Fernández-Lao C, Caro-Morán E, Morillas-Ruiz J, Galiano- astillo N, Díaz-Rodríguez L, Arroyo-Morales M. Aquatic exercise in a chest-high pool for hormone therapy-induced arthralgia in breast cancer survivors: a pragmatic controlled trial. Clin Rehabil. 2013 Feb;27(2):123-32.
- Cantarero-Villanueva I, Fernández-Lao C, Fernández-de-Las-Peñas C, López-Barajas IB, Del-Moral-Ávila R, de la-Llave-Rincón AI, Arroyo-Morales M. Effectiveness of water physical therapy on pain, pressure pain sensitivity, and myofascial trigger points in breast cancer survivors: a randomized, controlled clinical trial. Pain Med. 2012 Nov;13(11):1509-19.
- Cappiello J, Piazza C, Giudice M, De Maria G, Nicolai P. Shoulder disability after different selective neck dissections (levels II–IV versus levels II–V): a comparative study. Laryngoscope. 2005;115(2):259–63.
- Cheville AL, Tchou J. Barriers to rehabilitation following surgery for primary breast cancer. J SurgOncol 2007;95(5):409–418.
- Chrischilles EA, Riley D, Letuchy E, et al. Upper extremity disability and quality of life after breast cancer treatment in the Greater Plains Collaborative clinical research network. Breast Cancer Res Treat. 2019;175(3):675-689.
- Couch M, Lai V, Cannon T, Guttridge D, Zanation A, George J, Hayes DN, Zeisel S, Shores C. Cancer cachexia syndrome in head and neck cancer patients: part I.Diagnosis, impact on quality of life and survival, and treatment. Head Neck. 2007 Apr;29(4):401-11.
- De Groef A, Devoogdt N, Van der Gucht E, *et al*. EduCan trial: study protocol for a randomised controlled trial on the effectiveness of pain neuroscience education after breast cancer surgery on pain,physical, emotional and workrelated functioning. *BMJ Open* 2019;9:e025742.
- Epstein JB, Robertson M, Emerton S, Phillips N, Stevenson-Moore P. Quality of life and oral function in patients treated with radiation therapy for head and neck cancer. Head Neck. 2001 May;23(5):389-98.
- Fernández-Lao C, Cantarero-Villanueva I, Fernández-de-Las-Peñas C, del Moral-Ávila R, Castro-Sánchez AM, Arroyo-Morales M. Effectiveness of a multidimensional physical therapy program on pain, pressure hypersensitivity, and trigger points in breast cancer survivors: a randomized controlled clinical trial. Clin J Pain. 2012 Feb;28(2):113-21.
- Hojan K, Milecki P. Opportunities for rehabilitation of patients with radiation fibrosis syndrome. Reports of Practical Oncology and Radiotherapy. 2014;19:1-6.
- Johansson S, Svensson H, Denekamp J. Dose response and latency for radiation-induced fibrosis, edema, and neuropathy in breast cancer patients. Int J RadiatOncolBiolPhys 2002;52(5):1207–1219.
- Ling CC, Lui LY, So WK. Do educational interventions improve cancer patients' quality of life and reduce pain intensity? Quantitative systematic review. *J Adv Nurs* 2012;68:511–20.
- Lozano-Lozano M, Cantarero-Villanueva I, Martin-Martin L, Galiano-Castillo N, Sanchez MJ, Fernández-Lao C, Postigo-Martin P, Arroyo-Morales M. A Mobile System to Improve Quality of Life Via Energy Balance in Breast Cancer Survivors (BENECA mHealth): Prospective Test-Retest Quasiexperimental Feasibility Study. *JMIR Mhealth Uhealth*. 2019;7(6):e14136.
- Moseley GL, Butler DS. Fifteen years of explaining pain: the past, present, and future. *J Pain* 2015;16:807–13.
- National Comphrehensive Cancer Network. Head and neck cancers, version 1; 2016. Disponible en: https://www.nccn.org/professionals/ physician_gls/PDF/head-and-neck.pdf.
- Nijs J, Paul van Wilgen C, Van Oosterwijck J, *et al*. How to explain central sensitization to patients with 'unexplained' chronic musculoskeletal pain: practice guidelines. *Man Ther* 2011;16:413–8.

- Nijs J, Torres-Cueco R, van Wilgen CP, *et al*. Applying modern pain neuroscience in clinical practice: criteria for the classification of central sensitization pain. *Pain Physician* 2014;17:447–57.
- Ortiz-Comino L, Fernández-Lao C, Castro-Martín E, et al. Myofascial pain, widespread pressure hypersensitivity, and hyperalgesia in the face, neck, and shoulder regions, in survivors of head and neck cancer. *Support Care Cancer*. 2020;28(6):2891-2898.
- Ortiz-Piña M, Salas-Fariña Z, Mora-Traverso M, Martín-Martín L, Galiano-Castillo N, GarcíaMontes I, Cantarero-Villanueva I, Fernández-Lao C, Arroyo-Morales M, Mesa-Ruíz A, CastelloteCaballero Y, Salazar-Graván S, Kronborg L, Martín-Matillas M, Ariza-Vega P. A home-based telerehabilitation protocol for patients with hip fracture called @ctivehip. Res Nurs Heal. 2019
- Pauloski BR. Rehabilitation of dysphagia following head and neck cancer. Phys Med Rehabil Clin N Am. 2008;19(4):889-928).
- Rathod S, Livergant J, Klein J,Witterick I, Ringash J. A systematic review of quality of life in head and neck cancer treated with surgery with or without adjuvant treatment. Oral Oncol. 2015;51(10): 888–900.
- Sánchez-Jiménez A, Cantarero-Villanueva I, Molina-Barea R, Fernández-Lao C, Galiano-Castillo N, Arroyo-Morales M. Widespread pressure pain hypersensitivity and ultrasound imaging evaluation of abdominal area after colon cancer treatment. Pain Med. 2014 Feb;15(2):233-40.
- Shamley D, Lascurain-Aguirrebeña I, Oskrochi R, Srinaganathan R. Shoulder morbidity after treatment for breast cancer is bilateral and greater after mastectomy. Acta Oncol. 2012;51(8):1045-1053.
- Wyper GMA, Assunção R, Cuschieri S, Devleeschauwer B, Fletcher E, Haagsma JA, et al. Population vulnerability to COVID-19 in Europe: a burden of disease analysis. Arch Public Health. 2020;78:47.

4.3. HYPOTHESIS, RESEARCH QUESTION OR DESCRIPTIVE STUDY

The hypothesis of this project is that both a physical recovery program (therapeutic exercise and myofascial recovery techniques) in combination with the use of an educational app, and a physical recovery program (therapeutic exercise and myofascial recovery techniques), in combination with the traditional biomedical recommendations, will be effective in the treatment of pain and dysfunction of cancer patients after medical treatments, thus improving their quality of life. The physical recovery program in combination with the use of PaiNEd will represent a major improvement in this population. This improvement will be maintained after a follow-up period.

PROBLEM STATEMENT

The benefits of applying multimodal programs as a form of recovery in an emerging population that has overcome cancer are showing sufficient scientific evidence that justifies the appearance of new health services that respond to the needs of these patients during the recovery process. In this sense:

a) The higher incidence of the disease and the therapeutic success due to early diagnosis increase the number of people who survive cancer, although the secondary effects derived from the treatment must be attended by the health system.

b) There are no rehabilitation programs that have added Pain Neuroscience Education to the standardized and useful therapeutic proposals for this population group.

c) There is no information on how this type of rehabilitation services can reduce musculoskeletal disorders in this type of patients and improve the rest of the physical sequelae, and consequently their quality of life.

4.4. OBJECTIVES

OE1: Design and define the PaiNEd evaluation system.
In this first objective, through the "expertise" of the different aggregates, the specific contents of the assessment of pain and physical disability will be defined so that they are adapted with the greatest precision to the target population of this study.

OE2: Design and define the PaiNEd education system
Implementation of a system of recommendations based on the evidence currently available on education in the neuroscience of pain and on the results obtained in the monitoring phase of the system.

OE3: Develop the technology and technical architecture of the PaiNEd system
Technological development of the system in its initial version, fine-tuning of the system for its use in real clinical situations. Internal monitoring phase of the system.

OE4. Randomized controlled clinical trial. Effectiveness study
The objective will be to examine the effectiveness of a PNE tool as a supplement to a multimodal treatment program based on therapeutic exercise and myofascial recovery techniques to treat pain and dysfunction in breast cancer survivors compared to a multimodal treatment program combined with traditional biomedical information.

4.5. METHODOLOGY

Participants will be patients with breast cancer who have completed treatment and up to two years after completion. Patients will be recruited from the Medical Oncology and/or Radiation Oncology services of the Hospital Virgen de las Nieves and the Hospital de Parque Tecnológico de la Salud in Granada.

Inclusion criteria: a) Subjects over 18 years of age. b) Having undergone surgery and completed adjuvant treatment (radiotherapy and/or chemotherapy), from six months to two years ago, c) Not having active cancer, d) Having pain in the regions related to the tumor area (cervical and/or brachial and/or shoulder), pain ≥ 4 (VAS 0 to 10) for >4 weeks e) Present musculoskeletal or functional alterations in the previous regions.

Exclusion criteria: a) Identification by the research team of physical or mental impossibility to carry out the tests and tests of the study, b) Suffering from chronic pain or having suffered previous trauma to the spine, head, temporomandibular joint or upper limbs.

Study design: Experimental design with two intervention groups and a control group. Effectiveness study. The inclusion and exclusion criteria will be the same as for the previous phase.

GE1: multimodal rehabilitation program + PNE

GE2: multimodal rehabilitation program + traditional biomedical information

CG: control group (patient waiting list)

Sample size calculation. The estimation of the sample size and power for STUDY II have been determined for the main variable, pain measured through the Visual Analogue Scale (VAS) (Jensen, 1993). Based on a previous study (De Groef et al. 2017) and estimating that the patients in the intervention group would have a difference of 15mm or more in the VAS compared to the control group, differences of at least 5% could be detected with a power of 80% and an alpha level α 0.05. For this, a sample of 24 patients per group will be necessary, which will give a total of 72 breast cancer survivors, which will allow a maximum follow-up loss of 10%. To carry out this sample calculation, the Epidat 4.2 Software was used.

Randomization and blinding: Participants who meet the inclusion criteria will be randomized into each of the groups using a random number generation program (EPIDAT 4.2, Xunta de Galicia). The sequence will be placed in an opaque envelope sealed by a member external to the research and will be opened once the baseline assessment is completed, so that the assessment staff will be masked regarding the randomization of the participants, thus reducing the risk of bias during the valuation. The blinding of evaluators will be guaranteed by the fact that some members of the group are specialized in the treatment of cancer patients and others in their evaluation.

INTERVENTIONS

EXPERIMENTAL GROUP 1: MULTIMODAL REHABILITATION PROGRAM (THERAPEUTIC EXERCISE + MANUAL THERAPY). Participants will receive the intervention through participation in the physical recovery program (multimodal) with a duration of 8 weeks. Patients will receive three

weekly sessions on alternate days lasting 60 min. The sessions will consist of therapeutic exercise group work: a) warm-up (10 min aerobic work), b) active mobilizations (cervical/shoulder) c) strength work of the cervical and shoulder musculature + general conditioning work d) work of postural control e) stretching, relaxation. In addition, patients will receive a total of 4 individual manual therapy sessions (1 session every 2 weeks). Finally, patients will have access to the PaiNEd system before the start of the program (to prepare them for the treatment sessions) and during the development of the treatment program where they will enter pain levels and characteristics and receive a series of educational advice and recommendations for its improvement.

<u>EXPERIMENTAL GROUP 2: TREATMENT PROGRAM BY MANUAL THERAPY.</u> Participants will receive the intervention through participation in the physical recovery program (multimodal) with a duration of 8 weeks. Patients will receive three weekly sessions on alternate days lasting 60 min. The program will consist of the same treatment proposed for GE1, but patients will receive a dossier with traditional biomedical recommendations on pain management and disability associated with side effects of cancer treatment.

<u>CONTROL GROUP.</u> Waiting list of patients who have agreed to participate in the study. They will receive an information dossier with recommendations on pain control and dysfunction improvement (similar to what was worked on in the experimental group). These recommendations will be distributed at the beginning of the study (initial assessment) and they will be called at 8 weeks for the next collection of variables. All patients will be invited to participate in the experimental phase at the end of the control phase.

<u>RANDOMIZED CONTROLLED CLINICAL TRIAL OUTCOMES (OE4)</u>

The study variables will be evaluated at baseline, at the end of the intervention (2 months) and 6 months after its completion.

A questionnaire of sociodemographic variables will be provided at the beginning of the assessment. The clinical data will be taken from the electronic record by the participating health personnel.

<u>Pain and Function outcomes</u>

Pain: Visual Analogue Scale to assess pain in the neck, temporomandibular area and shoulder (Fernández-Lao et al. 2010) and Brief Pain Inventory (to assess quality, location and interference of pain. Galiano-Castillo et al. 2014)

Central Sensitization Inventory. Presence and degree of hypersensitivity to non-mechanical stimuli (Mayer et al. 2012).

Pressure pain thresholds (Algometry): It will be carried out in the points related to the cervical area and the shoulder girdle (C5-C6 joint, upper trapezius, levator scapulae, masseter, deltoids, sternoclavicular joint and a point at the tibialis anterior). (Fernández-Lao et al. 2010; 2011).

Pain-Related Catastrophizing: Pain Catastrophizing Scale: Used to assess pain-related catastrophic thinking (Sullivan et al. 1995).

Active shoulder and cervical spine range of motion: Using a goniometer and cervical inclinometer. (Cantarero-Villanueva et al. 2011)

<u>Quality of life and physical function outcomes</u>

Quality of life: general and specific cancer questionnaires (EORT QLQ-C30 + breast module [BR-23] (Singer et al. 2009).

Fatigue: Piper's Fatigue Questionnaire (Cantarero-Villanueva et al. 2014)

Functional capacity: Through the 6-minutes walking test (Laskin et al. 2007).

Manual grip strength: Using manual isometric dynamometry (España-Romero, 2010).

Evaluation of deep cervical musculature strength resistance: Using craniocervical flexion test and musculoskeletal ultrasound (Edmonston et al. 2008)

Body composition: Using an electrical bioimpedance analysis (Inbody 720) (Galiano-Castillo et al. 2014).

Heart rate variability: Through a Holter recorder (Fernández-Lao et al. 2011).

Inflammatory and stress response: Salivary biomarkers of Cortisol, IL6 and PCR. This determination will be made to assess the effect of the physical recovery program against the recommendations of the control group, following standardized procedures in the laboratory of the Faculty of Health Sciences. (Cantarero-Villanueva et al. 2012).

Statistical analysis

To analyze the results, the statistical program IBM SPSS 24.0 will be used. The Kolmogorov-Smirnov Test will be applied to verify the normal distribution of the variables (P>0.05). ROC curves will be included to determine the accuracy of the tests. Descriptive statistics for this study will use mean and standard deviation values along with 95% confidence intervals. The previous values of each follow-up variable will be compared through the T-Student test for independent samples. From the point of view of inferential statistics and to respond to the main objective of this study, an intention-to-treat analysis will be performed. A 3x3 ANOVA repeated measures model will be carried out using time as the intra-subject factor (baseline, post-intervention, follow-up) and intervention (GE1, GE2, control) as the inter-subject factor. The Bonferroni test will be used for post-hoc analysis. For the study on subgroups with possible differential results (elevated tumor stages, age, gender) they will be used as possible confounding variables in a subsequent multiple regression analysis. A statistical significance level of p-value less than 0.05 will be considered.

4.6. WORK PLAN

1º Year Activity	1 Month	2 Month	3 Month	4 Month	5 Month	6 Month	7 Month	8 Month	9 Month	10 Month	11 Month	12 Month
WP1												
WP2												
WP3												
2º Year Activity	13 Month	14 Month	15 Month	16 Month	17 Month	18 Month	19 Month	20 Month	21 Month	22 Month	23 Month	24 Month
WP3												
WP4												
3º Year Activity	25 Month	26 Month	27 Month	28 Month	29 Month	30 Month	31 Month	32 Month	33 Month	34 Month	35 Month	36 Month
WP4												
WP5												
WP6												
