# Supplementary material for: Persistent pain management in an oncology population through pain neuroscience education, a multimodal program: PaiNEd randomized clinical trial protocol
Source: PLoS One. 2023 Aug 15;18(8):e0290096. doi: 10.1371/journal.pone.0290096 (PMC10426993; doi:10.1371/journal.pone.0290096)
Supplement: S2 File — (PDF) [file pone.0290096.s004.pdf]

HOJA DE INFORMACIÓN AL PARTICIPANTE

Título del estudio: PaiNEd Study: EFECTIVIDAD DE UN SISTEMA DE E-HEALTH INTEGRADO EN UN PROGRAMA DE RECUPERACIÓN FÍSICA PARA EL TRATAMIENTO DEL DOLOR EN POBLACIÓN ONCOLÓGICA.

.

INVESTIGADOR PRINCIPAL/DEPARTAMENTO/EMAIL:

CAROLINA FERNÁNDEZ LAO/FISIOTERAPIA/carolinafl@ugr.es

CENTRO:

UNIDAD DE APOYO AL PACIENTE ONCOLOGICO (GRANADA)

1. INTRODUCCIÓN

Le informaremos acerca de un estudio de investigación, aprobado por el Comité de Ética, en el que está invitado a participar. Nuestra única intención es que reciba la información correcta y suficiente para que pueda evaluar y juzgar si desea o no participar en este estudio. Para hacer esto, lea esta Hoja de Información cuidadosamente. Puede consultar con las personas que considere apropiadas y aclararemos cualquier duda que pueda surgir.

El investigador y las entidades colaboradoras se comprometen a cumplir estrictamente la legislación nacional, regional y de la UE que cubre el uso de datos humanos con fines científicos.

Este proyecto está en concordancia con los Principios éticos fundamentales, incluidos los reflejados en la Carta de los Derechos Fundamentales de la Unión Europea[1] y las reglas éticas relevantes de H2020. Además, se aplican las siguientes normas internacionales:

Declaración de WMA de Helsinki[2]

Convención de Bioética de Oviedo[3]

---

[1] Charter of Fundamental Rights of the European Union, 2000/C 364/01, http://www.europarl.europa.eu/charter/pdf/text_en.pdf

[2] Declaration of Helsinki, http://www.who.int/bulletin/archives/79(4)373.pdf

[3] Convention for the Protection of Human Rights and Dignity of the Human Being with regard to the Application of Biology and Medicine: Convention on Human Rights and Biomedicine, https://rm.coe.int/CoERMPublicCommonSearchServices/DisplayDCTMContent?documentId=090000168007cf98

2.PARTICIPACIÓN VOLUNTARIA

Debe saber que su participación en este estudio es voluntaria, y puede decidir no participar o cambiar su decisión y retirar su consentimiento en cualquier momento. Su participación en el estudio en ningún caso representará una carga financiera adicional para usted.

3.DESCRIPCIÓN GENERAL DEL ESTUDIO

Este proyecto lo llevará a cabo el personal del grupo de investigación llamado CUIDATE y se desarrollará en la Unidad de Apoyo al Paciente Oncológico que surge del convenio establecido entre la Universidad de Granada (Instituto de Investigación Deporte y Salud) y el Servicio Andaluz de Salud. Dicho centro cuenta con los espacios y equipamiento necesarios para completar el programa de intervención y las pertinentes evaluaciones basal, post-tratamiento y tras el seguimiento. Somos un grupo de fisioterapeutas, terapeutas ocupacionales, médicos oncólogos, especialistas en actividad física y especialistas en medicina del deporte que tienen como objetivo mejorar la calidad de vida de los pacientes oncológicos. El grupo pretende evaluar los problemas que le preocupan y darle respuestas de forma individualizada.

Le estamos pidiendo que participe en este estudio porque podrá mejorar su calidad de vida tras haber sufrido cáncer. La quimioterapia forma parte, en la mayoría de los casos, del arsenal terapéutico que reciben los pacientes oncológicos junto con la radioterapia y la cirugía. Entre los efectos secundarios asociados el dolor y la disfunción pueden ser invalidantes para algunas personas y motivo de abono del tratamiento médico. Por ello, queremos ofrecerte una intervención basada en un programa de rehabilitación multimodal (acompañado de un programa de educación), sólo solo rehabilitación multimodal (más una serie de recomendaciones), o una serie de recomendaciones basadas en ejercicios para poder dar respuesta a las necesidades que le surgen después del proceso oncológico y para ayudarle en el camino de su recuperación. El propósito de este estudio es evaluar la efectividad de los tratamientos sobre la calidad de vida. Se está llevando a cabo esta investigación por dos razones fundamentales: son técnicas seguras, específicas para el dolor y la disfunción y coste-efectivas, y además porque no existen estudios que usen esta combinación de tratamientos, aunque de manera aislada ya han mostrado efectividad en otros síndromes de dolor crónico.

Aproximadamente 80 personas participarán en el estudio. Al tratarse de un estudio experimental, se realizarán dos grupos de intervención y un grupo control y ustedes podrán ser asignados de forma aleatoria a uno de los grupos.

Si usted decide participar en este estudio, tendrá que dar respuestas a una serie de cuestionarios sobre su salud y realizar test físicos para analizar la respuesta de su cuerpo al recibir los tratamientos descritos anteriormente.

Permanecerá en el estudio 32 semanas (8 meses) debido al seguimiento que se realizará durante este tiempo. Le realizaremos una valoración basal, al inicio del estudio, y dos valoraciones de seguimiento: una al finalizar la intervención y otra a los 6 meses.

4.BENEFICIOS Y RIESGOS DERIVADOS DE SU PARTICIPACIÓN EN EL ESTUDIO.

Los posibles beneficios de participar en el estudio son al menos los mismos que se obtienen al recibir intervención convencional sin participar en el estudio.

Le notificaremos sobre cualquiera nueva información que pueda afectar a su salud, bienestar o interés por continuar en el estudio.

Con su participación puede ayudarnos a mejorar la información existente, lo que nos permite mejorar la asistencia a las personas que pasan por un proceso similar al suyo. Obtendrá un informe detallado de todas las evaluaciones llevadas a cabo una vez finalizado el proyecto, que verá la evolución de su salud a través de su tratamiento. Por otro

lado, también recibirá pautas sobre manejo del dolor y bienestar psicológico que puede seguir una vez que el proyecto finalice, independientemente del grupo de estudio al que pertenezca.

5. PROTECCIÓN DE DATOS

El procesamiento, la comunicación y la transferencia de los datos personales de todos los participantes se realizarán de conformidad con las reglamentaciones locales, nacionales e internacionales. De acuerdo con la legislación antes mencionada, puede ejercer sus derechos de acceso, modificación, oposición y cancelación de datos, para lo cual debe contactar a su persona de estudio a cargo.

A partir del 25 de mayo de 2018 es de plena aplicación la nueva legislación en la UE sobre datos personales, en concreto el Reglamento (UE) 2016/679 del Parlamento europeo y del Consejo de 27 de abril de 2016 de Protección de Datos (RGPD). Por ello, es importante que conozca la siguiente información.

- Además de los derechos que ya conoce (acceso, modificación, oposición y cancelación de datos) ahora también puede limitar el tratamiento de datos que sean incorrectos, solicitar una copia o que se trasladen a un tercero (portabilidad) los datos de usted ha facilitado para el estudio. Para ejercitar sus derechos, diríjase al investigador principal del estudio. Le recordamos que los datos no se pueden eliminar aunque deje de participar en el ensayo para garantizar la validez de la investigación. Así mismo tiene derecho a dirigirse a la Agencia de Protección de Datos si no quedara satisfecho/a.
- Tanto el Centro como el Promotor son responsables respectivamente del tratamiento de sus datos y se comprometen a cumplir con la normativa de protección de datos en vigor. Los datos recogidos para el estudio estarán identificados mediante un código, de manera que no se incluya información que pueda identificarle, y sólo su médico del estudio/colaboradores podrá relacionar dichos datos con usted y con su historia clínica. Por lo tanto, su identidad no será revelada a ninguna otra persona salvo a las autoridades sanitarias, cuando así lo requieran o en casos de urgencia médica. Los Comités de Ética de la Investigación, los representantes de la Autoridad Sanitaria en materia de inspección y el personal autorizado por el Promotor, únicamente podrán acceder para comprobar los datos personales, los procedimientos del estudio clínico y el cumplimiento de las normas de buena práctica clínica (siempre manteniendo la confidencialidad de la información).
- El Investigador y el Promotor están obligados a conservar los datos recogidos para el estudio al menos hasta 25 años tras su finalización. Posteriormente, su información personal solo se conservará por el centro para el cuidado de su salud y por el promotor para otros fines de investigación científica si usted hubiera otorgado su consentimiento para ello, y si así lo permite la ley y requisitos éticos aplicables.
- Si realizáramos transferencia de sus datos codificados fuera de la UE a las entidades de nuestro grupo, a prestadores de servicios o a investigadores científicos que colaboren con nosotros, los datos del participante quedarán protegidos con salvaguardas tales como contratos u otros mecanismos por las autoridades de protección de datos.

6. COMPENSACION ECONÓMICA

El investigador del estudio es responsable de gestionar el financiamiento de la misma. El sujeto no recibirá ninguna remuneración. La participación en el estudio no implicará un costo adicional.

7. OTRA INFORMACIÓN RELEVANTE

El grupo de investigación le comunicará lo antes posible cualquier información nueva relevante para el estudio que pueda afectar su disposición a participar en el estudio, que se descubra durante su participación.

Si decide retirar su consentimiento para participar en este estudio, no se agregarán nuevos datos a la base de datos y puede requerir la destrucción de datos anteriores.

También debe saber que puede ser excluido del estudio si los investigadores del estudio lo consideran apropiado, ya sea por razones de seguridad, por cualquier ocurrencia que ocurra o porque consideren que no está cumpliendo con los procedimientos establecidos. En cualquier caso, recibirá una explicación adecuada del motivo de su retirada del

estudio. Al firmar el formulario de consentimiento informado, usted acepta cumplir con los procedimientos de estudio que se han establecido.

CONSENTIMIENTO INFORMADO – CONSENTIMIENTO POR ESCRITO DEL PACIENTE

PaiNEd Study: EFECTIVIDAD DE UN SISTEMA DE E-HEALTH INTEGRADO EN UN PROGRAMA DE RECUPERACIÓN FÍSICA PARA EL TRATAMIENTO DEL DOLOR EN POBLACIÓN ONCOLÓGICA.

Yo (Nombre y Apellidos):..

- He leído el documento informativo que acompaña a este consentimiento (Información al Paciente)
- He podido hacer preguntas sobre el estudio "**PaiNEd Study: EFECTIVIDAD DE UN SISTEMA DE E-HEALTH INTEGRADO EN UN PROGRAMA DE RECUPERACIÓN FÍSICA PARA EL TRATAMIENTO DEL DOLOR EN LA POBLACIÓN ONCOLÓGICA**"
- He recibido suficiente información sobre el estudio ""**PaiNEd Study: EFECTIVIDAD DE UN SISTEMA DE E-HEALTH INTEGRADO EN UN PROGRAMA DE RECUPERACIÓN FÍSICA PARA EL TRATAMIENTO DEL DOLOR EN LA POBLACIÓN ONCOLÓGICA**" I have spoken with the reporting healthcare profesional ....
- Comprendo que mi participación es voluntaria y soy libre de participar o no en el estudio.
- Se me ha informado que todos los datos obtenidos en este estudio serán confidenciales y se tratarán conforme establece la Ley Orgánica de Protección de Datos de Carácter Personal 15/99.
- Se me ha informado de que la donación/información obtenida sólo se utilizará para los fines específicos del estudio.
- **Deseo** ser informado/a de mis datos genéticos y otros de carácter personal que se obtengan en el curso de la investigación, incluidos los descubrimientos inesperados que se puedan producir, siempre que esta información sea necesaria para evitar un grave perjuicio para mi salud o la de mis familiares biológicos.
  Si No

Comprendo que puedo retirarme del estudio:

- Cuando quiera
- Sin tener que dar explicaciones
- Sin que esto repercuta en mis cuidados médicos

Presto libremente mi conformidad para participar en el *proyecto titulado "***PaiNEd Study: EFECTIVIDAD DE UN SISTEMA DE E-HEALTH INTEGRADO EN UN PROGRAMA DE RECUPERACIÓN FÍSICA PARA EL TRATAMIENTO DEL DOLOR EN LA POBLACIÓN ONCOLÓGICA**"

Firma del paciente	Firma del profesional
(o representante legal en su caso)	sanitario informador
Nombre y apellidos:……………….	Nombre y apellidos: ………..
Fecha: …………………………….	Fecha: ……………………….

REVOCACIÓN DEL CONSENTIMIENTO INFORMADO PARA LA PARTICIPACIÓNEN

EL PROYECTO DE INVESTIGACIÓN

Yo, D./Dª ……………………………………………………, con DNI/NIE……………………………………, como (marcar lo que proceda): SUJETO/ REPRESENTANTE LEGAL, revoco libremente el consentimiento informado para la participación en el proyecto de investigación firmado en el presente documento.

* En este caso, al tener el sujeto participante un impedimento para escribir, la revocación del consentimiento se realiza de forma oral en presencia del testigo D./Dª ……………………………………, con DNI/NIE……………………., que firma a continuación

Firma del sujeto/Representante legal/Testigo	Firma del investigador y Nº de colegiado

PARTICIPANT'S INFORMATION SHEET

TITLE OF THE STUDY: PaiNEd Study: EFFECTIVENESS OF AN E-HEALTH SYSTEM INTEGRATED IN A PHYSICAL RECOVERY PROGRAM FOR THE TREATMENT OF PAIN IN CANCER SURVIVORS. PaiNEd STUDY.

PRINCIPAL INVESTIGATOR/DEPARTMENT/EMAIL:

CAROLINA FERNÁNDEZ LAO/PHYSIOTHERAPY

carolinafl@ugr.es

CENTER:

UNIDAD DE APOYO AL PACIENTE ONCOLOGICO (GRANADA)

1. INTRODUCTION

We will inform you about a research study, approved by the Ethics Committee, in which you are invited to participate. Our only intention is that you receive the correct and sufficient information so that you can evaluate and judge whether or not you want to participate in this study. To do this, please read this Information sheet carefully. You can consult with the people you consider appropriate and we will clarify any doubt that may arise.

The researcher and collaborating entities commit to strictly comply with national, regional and EU legislation covering the use of human data for scientific purposes.

This project is in accordance with the Fundamental Ethical Principles, including those reflected in the Charter of Fundamental Rights of the European Union[1] and the relevant ethical rules of H2020. In addition, the following international standards apply:

WMA Declaration of Helsinki[2]

Convención de Bioética de Oviedo[3]

---

[1] Charter of Fundamental Rights of the European Union, 2000/C 364/01, http://www.europarl.europa.eu/charter/pdf/text_en.pdf

[2] Declaration of Helsinki, http://www.who.int/bulletin/archives/79(4)373.pdf

[3] Convention for the Protection of Human Rights and Dignity of the Human Being with regard to the Application of Biology and Medicine: Convention on Human Rights and Biomedicine, https://rm.coe.int/CoERMPublicCommonSearchServices/DisplayDCTMContent?documentId=090000168007cf98

2.VOLUNTARY PARTICIPATION

You should know that your participation in this study is voluntary, and you can decide not to participate or change your decision and withdraw your consent at any time. Your participation in the study will in no way represent an additional financial burden for you.

3.GENERAL DESCRIPTION OF THE STUDY

This project will be carried out by the staff of the research group called CUIDATE and will be developed in the Unidad de Apoyo al Paciente Oncológico that arises from the agreement established between the University of Granada (Sports and Health Research Institute) and the Andalusian Health Service. This center has enough room and equipment to complete the intervention program and the relevant baseline, post-treatment and follow-up assessments. We are a group of physiotherapists, occupational therapists, medical oncologists, physical activity specialists and sports medicine specialists who aim to improve the quality of life of cancer patients. The group aims to assess the problems that concern you and give you answers on an individual basis.

We are asking you to participate in this study because it may improve your quality of life after having cancer. Chemotherapy is part, in most cases, of the therapeutic tools that cancer patients receive along with radiotherapy and surgery. Among the associated side effects, pain and dysfunction can be disabling for some people and a reason for paying for medical treatment. For this reason, we want to offer you an intervention based on a multimodal rehabilitation program (accompanied by an education program), only multimodal rehabilitation (plus a series of recommendations), or a series of recommendations based on exercises in order to respond to the needs that arise after the oncological process and to help you on the road to recovery. The purpose of this study is to assess the effectiveness of these treatments on quality of life. This research is being carried out for two fundamental reasons: they are safe, specific for pain and dysfunction, and cost-effective techniques, and also because there are no studies that use this combination of treatments, although in isolation they have already shown effectiveness in other chronic pain syndromes.

Approximately 80 patients will participate in the study. As it is an experimental study, there will be two intervention groups and a control group and you may be randomly assigned to one of the groups. If you decide to participate in this study, you will have to answer a series of questionnaires about your health and take physical tests to analyze your body's response to receiving the treatments described above.

You will remain in the study for 32 weeks (8 months) due to follow-up during this time. We will perform a baseline assessment, at the beginning of the study, and two follow-up assessments, one at the end of the intervention and another one at 6 months of the study.

4.BENEFITS AND RISKS DERIVED FROM YOUR PARTICIPATION IN THE STUDY

The potential benefits of participating in the study are at least the same as those obtained from receiving conventional intervention without participating in the study.

We will notify you of any new information that may affect your health, well-being, or interest in continuing in the study.

With your participation you can help us improve existing information, which allows us to better assist people who are going through a process similar to yours. You will obtain a detailed report of all the assessments carried out once the project is finished, which will show the evolution of your health through your treatment.

On the other hand, you will also receive guidelines on pain management and psychological well-being that you can follow after the project ends, regardless of which study group you belong.

5. DATA PROTECTION

The processing, communication and transfer of personal data of all participants will be carried out in accordance with local, national and international regulations. In accordance with the aforementioned legislation, you can exercise your rights of access, modification, opposition and cancellation of data, for which you must contact the investigator in charge.

As of May 25, 2018, the new EU legislation on personal data is fully applicable, specifically Regulation (EU) 2016/679 of the European Parliament and of the Council of 27 April 2016 on Data Protection (GDPR). Therefore, it is important that you know the following information.

- In addition to the rights you already know (access, modification, opposition and cancellation of data) you can now also limit the processing of data that is incorrect, transfer to a third party (portability) the data you have provided for the study. To exercise your rights, contact the principal investigator of the study. We remind you that the data cannot be deleted even if you stop participating in the trial to ensure the validity of the research. Likewise, you have the right to contact the Data Protection Agency if you are not satisfied.
- Both the Center and the Promoter are respectively responsible for the processing of your data and commit to comply with the data protection regulations in force. The data collected for the study will be identified by a code, so that information that could identify you is not included, and only your study doctor/collaborators will be able to relate this data to you and your medical history. Therefore, your identity will not be revealed to any other person except the health authorities, when required or in cases of medical urgency. The Research Ethics Committees, the representatives of the Health Authority in inspection and the personnel authorized by the Sponsor, may only access to verify the personal data, the clinical study procedures and compliance with the standards of good clinical practice. (always maintaining the confidentiality of the information).
- The Investigator and the Promoter are obliged to keep the data collected for the study for at least 25 years after its completion. Subsequently, your personal information will only be retained by the health care center and the promoter for other scientific research purposes if you have given your consent to do so, and if permitted by applicable law and ethical requirements
- If we transfer your encrypted data outside the EU to our group entities, service providers or scientific researchers who collaborate with us, the participant's data will be protected with safeguards such as contracts or other mechanisms by data protection authorities. data.

6. FINANCIAL COMPENSATION

The study investigator is responsible for managing the financing of the study. The subject will not receive any remuneration. Participation in the study will not imply an additional cost.

7. OTHER RELEVANT INFORMATION

The research group will notify you as soon as possible of any new information relevant to the study that may affect your willingness to participate in the study, discovered during your participation.

If you decide to withdraw your consent to participate in this study, no new data will be added to the database and may require the destruction of old data.

You should also know that you can be excluded from the study if the study researchers consider it appropriate, either for safety reasons, for any occurrence that occurs, or because they consider that you are not complying with the established procedures. In any case, you will receive an adequate explanation of the reason for your withdrawal from the study. By signing the informed consent form, you agree to comply with the study procedures that have been established.

INFORMED CONSENT _ PATIENT WRITTEN CONSENT

PaiNEd Study: EFFECTIVENESS OF AN E-HEALTH SYSTEM INTEGRATED IN A PHYSICAL RECOVERY PROGRAM FOR THE TREATMENT OF PAIN IN CANCER SURVIVORS.

I (Name and Surnames):..

- I have read the informative document that comes along with this consent (Patient Information)
- I was able to ask questions about the study" **PaiNEd Study: EFFECTIVENESS OF AN E-HEALTH SYSTEM INTEGRATED IN A PHYSICAL RECOVERY PROGRAM FOR THE TREATMENT OF PAIN IN CANCER SURVIVORS.** "
- I have received enough information about the study: **PaiNEd Study: EFFECTIVENESS OF AN HEALTH SYSTEM INTEGRATED IN A PHYSICAL RECOVERY PROGRAM FOR THE TREATMENT OF PAIN IN CANCER SURVIVORS.** I have spoken with the reporting healthcare profesional ....
- I understand that my participation is voluntary and I am free to participate or not in the study.
- I have been informed that all the data obtained in this study will be confidential and will be treated in accordance with the Organic Law on Protection of Personal Data 15/99
- I have been informed that the donation/information obtained will only be used for the specific purposes of the study.
- I wish to be informed of my genetic data and other personal data obtained in the course of the research, including unexpected discoveries that may occur, whenever this information is necessary to avoid serious harm to my health or that of my biological relatives.

Yes No

I understand that I can withdraw the study:

- Whenever I want
- Without giving an explanation
- Without this affecting my medical care

I freely give my consent to participate in the study titled: **PaiNEd Study: EFFECTIVENESS OF AN HEALTH SYSTEM INTEGRATED IN A PHYSICAL RECOVERY PROGRAM FOR THE TREATMENT OF PAIN IN CANCER SURVIVORS ONCOLÓGICA.**

Patients' signature

(or legal representative if applicable)

Health professional's signature

health informant

Name and Surnames: ……………….

Date: …………………………………..

Name and Surnames: ……………….

Date: …………………………………..

REVOCATION OF INFORMED CONSENT TO PARTICIPATE IN THE RESEARCH PROJECT	
I…………………………….., With ID …………………………. As (check as appropriate):<br>SUBJECT / LEGAL REPRESENTATIVE, I freely revoke the informed consent for participation in the project of investigation signed in the present document<br><br>* In this case, since the participating subject has an impediment to write, the revocation of consent is performed orally in the presence of a witness. Mr. / Mrs: ……………, with ID: ………………. That signs below.	
**Signature of Subject/<br>Legal Representative/Witness**<br><br><br><br>Date:	**Researcher's signature and collegiate number**<br><br><br><br>Date:
